# Dynamic propeller conformation for the unprecedentedly high degree of chiral amplification of supramolecular helices†
†Electronic supplementary information (ESI) available: Synthesis, analytical data and Fig. S1–S34. See DOI: 10.1039/c6sc02814d



**DOI:** 10.1039/c6sc02814d

**Published:** 2016-07-12

**Authors:** Taehoon Kim, Tadashi Mori, Takuzo Aida, Daigo Miyajima

**Affiliations:** a RIKEN Center for Emergent Matter Science , 2-1 Hirosawa , Saitama 351-0198 , Wako , Japan . Email: daigo.miyajima@riken.jp; b Department of Chemistry and Biotechnology , School of Engineering , The University of Tokyo , 7-3-1 Hongo, Bunkyo-ku , Tokyo 113-8656 , Japan; c Department of Applied Chemistry , Graduate School of Engineering , Osaka University , 2-1 Yamada-oka , Suita , Osaka 565-0871 , Japan

## Abstract

An unprecedentedly high degree of chiral amplification of supramolecular helices in a sergeants and soldiers system was realized using a propeller-shaped molecule, triphenylamine (TPA), as the monomer. One sergeant controlled the handedness of 500 soldiers in supramolecular helices. We further demonstrated that a TPA derivative could switch its role from sergeant to soldier and *vice versa* depending on its partners. These achievements could be realized using the dynamic propeller conformation of TPA and provide new insights into supramolecular assemblies and the supramolecular chiral amplification of helices.

## Abstract 2

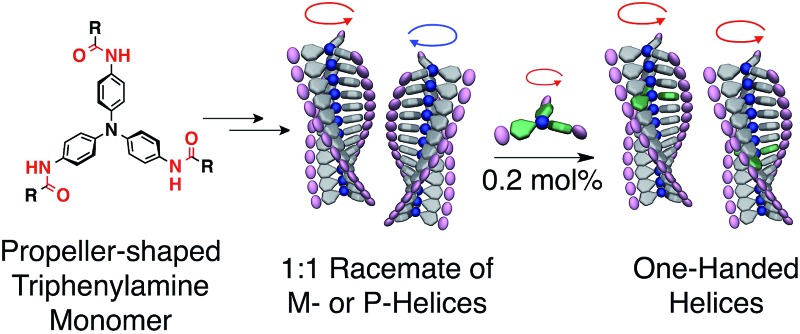
Unprecedentedly high degree of chiral amplification using dynamic propeller conformation of triphenylamine.

## Introduction

The helix has attracted scientists owing to its structural beauty, its vital roles in biological systems,^1^ and its potential for practical applications, such as chiral separation^2^ or asymmetric catalysis.^3^ The helix is intrinsically chiral and is always either left- or right-handed. To fully exploit the potential of helices,^1–3^ it is important to selectively synthesize or isolate helices with a single handedness. However, left- and right-handed helices are enantiomers, and their thermodynamic stabilities are identical. Hence, to selectively form one-handed helices, it is necessary to differentiate their stabilities kinetically or thermodynamically. For this purpose, a well-established method is the employment of chiral auxiliaries for racemic helices at the molecular and supramolecular levels.^4^ Owing to the introduction of other chiral sources, left- and right-handed helices become diastereomers of each other, and consequently, one of the helical structures becomes thermodynamically preferred. However, the presence of chiral auxiliaries inevitably alters the conformation of the helices and their physical properties.^4e,5^ Therefore, it is desirable to control the handedness of helices using a minimal amount of chiral auxiliaries *via* chiral amplification.

As represented by the pioneering works of Okamoto^6^ and Yashima,^7^ one-handed helices consisting of only achiral monomers have been successfully isolated, even in the absence of a chiral source.^8^ The helical polymers memorize the induced handedness by the action of chiral sources, even after the removal of the chiral sources. However, most reported helical polymers and supramolecular helices are dynamic and undergo racemization after removal of the chiral sources.^4^ In 1989, Green and his colleagues demonstrated the first chiral amplification system for helices in which a one-handed helical conformation of dynamic helices could be obtained by copolymerization of the achiral monomers with a small fraction of the chiral monomers.^9^ Because the chiral monomers dictate the handedness of the helices, which mainly consist of achiral monomers, this system is referred to as the sergeants (chiral monomers) and soldiers (achiral monomers) principle. This concept was later proven to be valid even for supramolecular helices.^10*a*^ Thanks to the systematic study by Meijer and coworkers using benzene-1,3,5-tricarboxamides (BTA) derivatives, now we have deep insights on the mechanism and important parameters for chiral amplifications in supramolecular helices.^10^ However, the degree of chiral amplification in the sergeants and soldiers system is still at most approximately 100 (one sergeant per 100 soldiers) for both dynamic helical polymers and supramolecular helices.^9,10d,11^ Because the concept of sergeants and soldiers is widely used for various helices,^4^ it is desirable to further extend this limit for both fundamental science and practical applications.

**Fig. 1:**
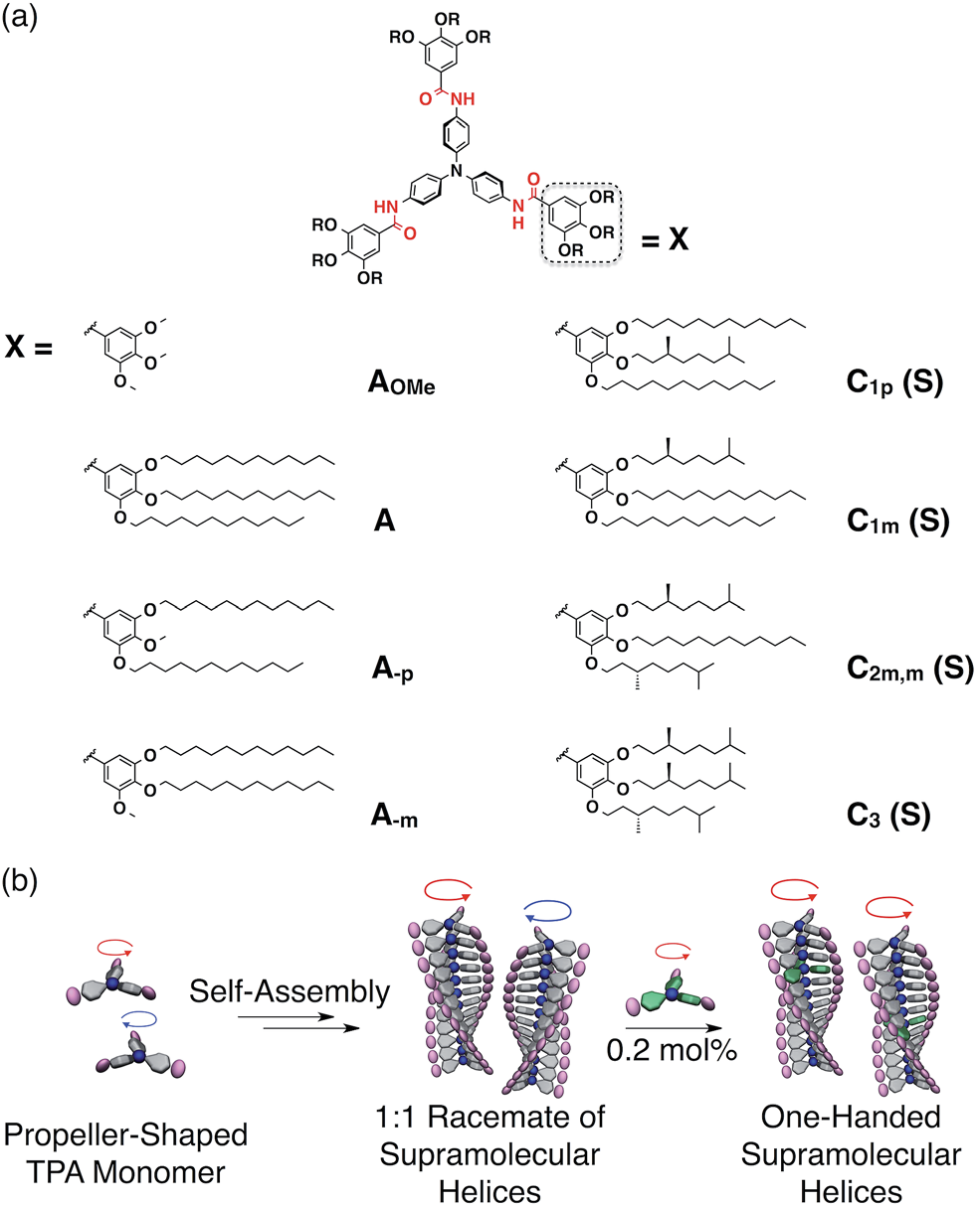
(a) Chemical structures of propeller-shaped triphenylamine (TPA) derivatives. (b) Schematic illustration of the self-assembly of TPA monomers into supramolecular helices and chiral amplification through the sergeants and soldiers principle. The green TPA monomer corresponds to a sergeant.

Here, we report an unprecedentedly high degree of amplification of supramolecular chirality in a sergeants and soldiers system using a propeller-shaped aromatic core in which only one sergeant controls the helicity of 500 soldiers in a supramolecular helix, on average (Fig. 1). Furthermore, we have substantiated the hierarchy of the sergeant molecules for the sergeants and soldiers systems.

## Result and discussion

**Fig. 2:**
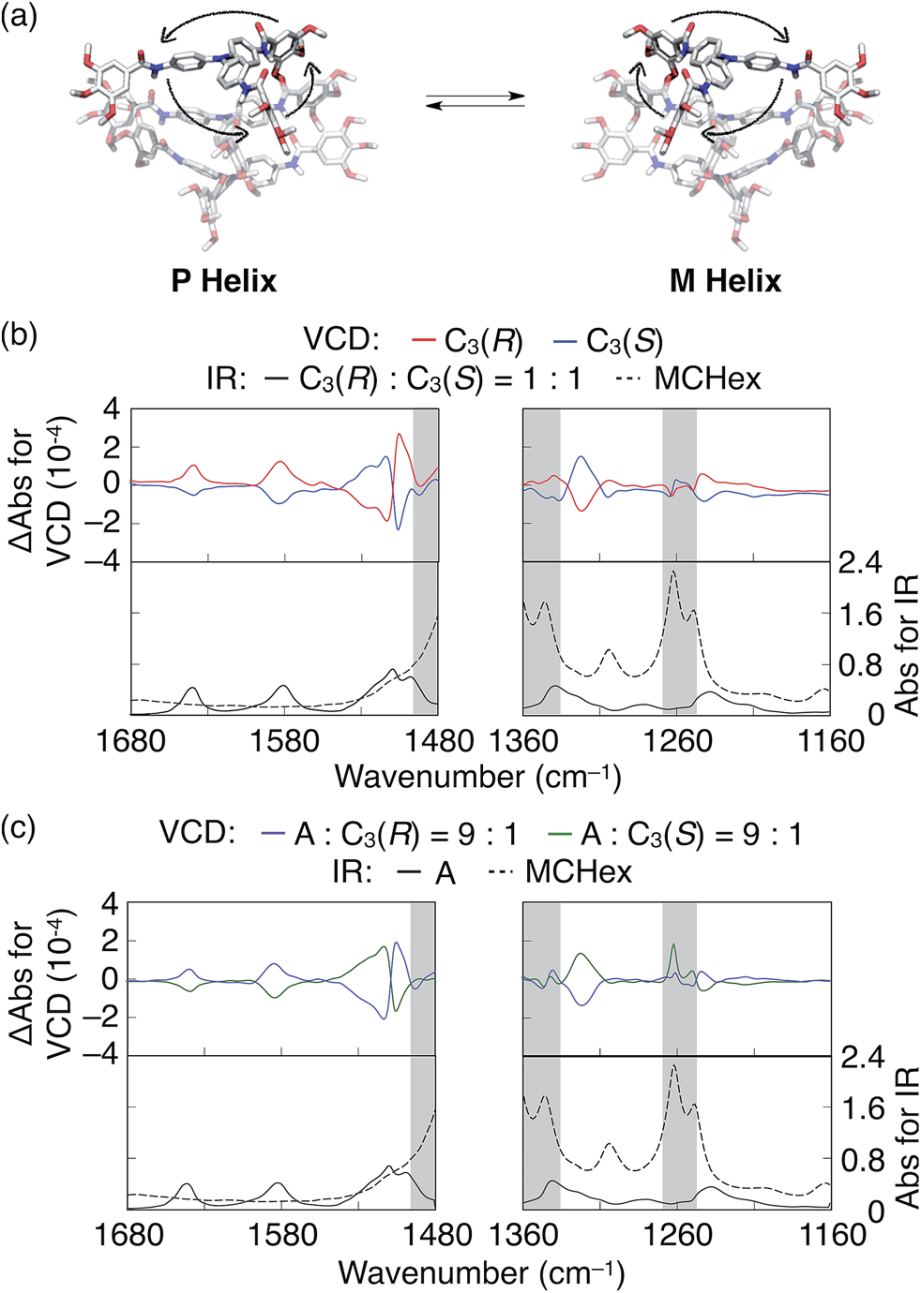
(a) Schematic representation of a trimeric **A_OMe_** whose structural optimization was performed at the DFT-D3(BJ)-TPSS/def2-TZVP level followed by frequency calculation at the same level. (b and c) VCD (upper) and IR (lower) spectra at 20 °C for 5 mM (b) **C_3_(*R*)** and **C_3_(*S*)** and of (c) **A** with 10 mol% **C_3_(*R*)** and **A** with 10 mol% **C_3_(*S*)** in methylcyclohexane (MCHex). The dashed line corresponds to the IR spectra of MCHex.

**Fig. 3:**
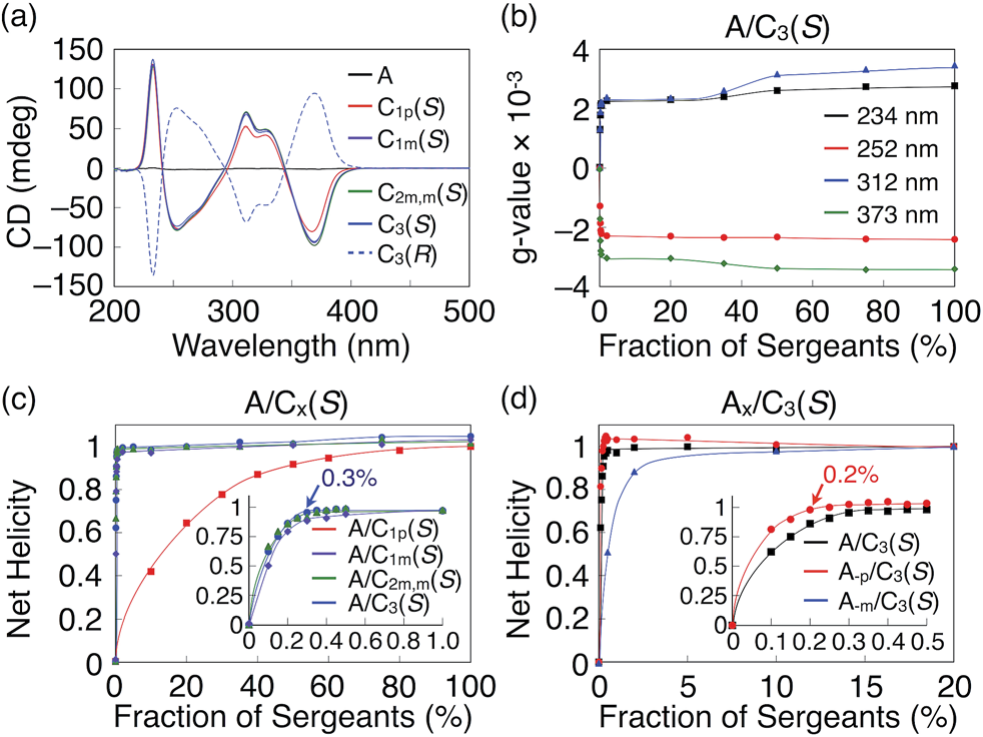
(a) CD spectra of **A**, **C_1p_(*S*)**, **C_1m_(*S*)**, **C_2m,m_(*S*)**, **C_3_(*S*)** and **C_3_(*R*)** in cyclohexane. (b) Plots of the *g*-values against the fraction of the sergeants for the mixture of **A**/**C_3_(*S*)** at 234, 252, 312 and 373 nm. (c) Plots of the net helicity against the fraction of the sergeants for the mixtures of **A**/**C_1p_(*S*)**, **A**/**C_1m_(*S*)**, **A**/**C_2m,m_(*S*)** and **A**/**C_3_(*S*)**. (d) Plots of the net helicity against the fraction of the sergeants for the mixtures of **A**/**C_3_(*S*)**, **A_-p_**/**C_3_(*S*)** and **A_-m_**/**C_3_(*S*)**. The total TPA concentration and the experimental temperature were 30 μM and 25 °C, respectively, for all measurements.

Recently, Giuseppone and coworkers have intensively investigated the self-assembling behaviours of triphenylamine (TPA) derivatives.^12^ In particular, they have suggested that TPA derivatives having amide groups in its side chains self-assemble into supramolecular helices and TPA derivatives selectively adopt (*P*)- or (*M*)-propeller chirality according to the handedness of the supramolecular helices in which they are assembled.^12*c*^ Inspired by these pioneering works, we wondered how such propeller chirality of TPA would affect the degree of chiral amplification. We then first performed a density functional theory (DFT) computational study^13^ to predict the optimal conformation of trimeric **A_OMe_** (Fig. 1a), a simplified model compound consisting of TPA as the core and three methoxylated benzamide groups. As expected,^12*c*^ the DFT calculation results indicated that **A_OMe_** forms a helical trimer *via* intermolecular hydrogen bonding of the amides and that all TPA cores adopt the same propeller chirality (Fig. 2a). We then synthesized similar TPA derivatives (Fig. 1a) and investigated their self-assembly behavior in cyclohexane. Dynamic light scattering (DLS) measurements suggested that **A** was molecularly dispersed at 75 °C and formed supramolecular aggregates at low temperature (25 °C, Fig. S19a†). Although the absorption spectra of **A** at 75 °C and 25 °C were similar (Fig. S19b†), a characteristic valley in the absorption spectra at approximately 315 nm appeared at 55 °C during the cooling process, suggesting the formation of supramolecular aggregates of **A** started at 55 °C (Fig. S19c†). Tapping-mode atomic force microscopy (AFM) measurements of the air-dried sample on a silicon wafer revealed that **A** forms one-dimensional supramolecular polymers (Fig. S20†), possibly by connecting *via* intermolecular hydrogen bonding and π–π interactions. The CD spectrum of **C_3_(*S*)** in cyclohexane exhibited characteristic exciton splitting at 312 nm and 328 nm (Fig. 3a, solid blue line), whereas **C_3_(*R*)** displayed the mirror-image CD spectrum under the same conditions, indicating the formation of one-handed supramolecular helices (Fig. 3a, dashed blue line). Based on the DFT calculations and the similarity between the absorption spectra of **A** and **C_3_(*S*)** in their assembled states (Fig. 3a and S21a†), we concluded that **A** also formed supramolecular helices, although they were a racemic mixture of left- and right-handed helices. Note that some TPA derivatives having amide groups are known to form lateral interaction between supramolecular helices.^12d,g^ However, due to the presence of multiple long alkyl-chains around amide groups, such lateral interactions are supposed to hardly happen in our system.

To determine whether the TPA core selectively adopts a supramolecular helices, we performed vibrational circular dichroism (VCD) measurements of methylcyclohexane solutions of **C_3_(*R*)** and **C_3_(*S*)**.^14^ As shown in Fig. 2b, **C_3_(*R*)** and **C_3_(*S*)** exhibited clear mirror-image spectra, with the exception of a few wavenumber ranges highlighted in gray in which the solvent absorption interference was not negligible. The VCD peaks between 1639 and 1584 cm^–1^ correspond to the stretching vibration of the C=O bond and the bending vibration of the N–H bond, respectively (Fig. S22†),^14b,15^ suggesting that the amide groups adopt a helical array in the assembled state. Moreover, the absorption bands with a strong Cotton effect at approximately 1500 cm^–1^ correspond to the stretching vibration and the side chains.^15,16^ The peaks at approximately 1322 and 1242 cm^–1^ correspond to the C–N stretching vibrational bands of the outer amine linked with TPA and the central amine present in TPA, respectively.^15,16^ The VCD data strongly suggest that the propeller chiralities of the TPA monomers are linked with the handedness of the supramolecular helices. However, based on these VCD data alone, it is difficult to exclude the possibility that the propeller chirality of TPA is decided by the side-chain chirality, independent of the handedness of the helices. Hence, we performed VCD experiments of a sergeants and soldiers system of **A**/**C_3_(*R*)** and **A**/**C_3_(*S*)** at a mole ratio of 9 to 1. We obtained VCD spectra nearly identical to those of **C_3_(*R*)** or **C_3_(*S*)** alone, indicating that TPA adopts a single propeller chirality according to the helicity of the supramolecular helices (Fig. 2b and c).

Next we investigated the degree of chiral amplification using **A** and **C_3_(*S*)** and found that one-handed helices of **A** could be obtained by mixing an unusually small fraction of **C_3_(*S*)** with **A**. In other words, the chiral information of the side chains of **C_3_(*S*)** was efficiently amplified into the handedness of the supramolecular helices of **A**. To accurately estimate the degree of chiral amplification of the mixed system of **A**/**C_3_(*S*)**, we performed the sergeants and soldiers experiments using various fractions of **C_3_(*S*)** (Fig. S23†). As shown in Fig. S23c,† the observed *g-*values varied depending on the fraction of **C_3_(*S*)** without noticeable changes in the CD spectral shapes. By plotting the *g*-values of the peak tops at 234, 252, 312, and 373 nm, we detected minor discontinuous changes of the *g*-values at an approximately 40 mol% fraction of **C_3_(*S*)** (Fig. 3b). The same feature was observed in the plots of the electronic absorption at the same wavelengths (Fig. S23d†). Although the electronic absorption spectra of **A** and **C_3_(*S*)** were nearly identical, the peak tops of **C_3_(*S*)** were slightly red-shifted by 1–2 nm on average (Fig. S21a†), indicating that the helical conformations of the supramolecular helices of **A** and **C_3_(*S*)** are slightly different. Hence, we assumed that co-assemblies of **A** and **C_3_(*S*)** adopt a helical conformation similar to that of either the homo-assembly of **A** or that of **C_3_(*S*)** and that the transition of this helical conformation occurs at an approximately 40 mol% fraction of **C_3_(*S*)**, resulting in the observed discontinuous changes in the *g*-values. Therefore, in the following studies, we used the *g*-value at a 20 mol% fraction of **C_3_(*S*)** as the value for the one-handed supramolecular helices of **A**. To clarify the magnitude of the changes in the *g*-values, we used net helicity as a probe for the degree of chiral amplification.^17^ As shown in the inset of Fig. 3c, the net helicity was saturated by addition of 0.3 mol% fraction of the sergeant. A single **C_3_(*S*)** manipulated the handedness of the supramolecular helices of 333 molecules of **A** on average. At this high degree of chiral amplification, the average length of the supramolecular helices may affect the result. However, the degree of chiral amplification of this system exhibited no change, even at a 30-fold diluted concentration (Fig. S24†), suggesting that the average length of the supramolecular helices are enough long not to limit the degree of chiral amplification.^10*a*^ Previously, 1 mol% sergeants was the minimum fraction to fully bias the handedness of supramolecular helices of soldiers.^4b,e,10d,11^ Hence, our TPA system exceeds the limit of the conventional sergeants and soldiers system.

Systematic studies of chiral amplification^10,18^ have revealed that two important parameters, mismatch penalty (MMP) and helical reversal penalty (HRP), govern the degree of chiral amplification in sergeant and soldier systems. The MMP is an energetic penalty incurred by a system when chiral sergeants are incorporated into non-preferred helices, whereas the HRP is the energetic barrier that maintains the handedness of supramolecular helices. In general, as these two parameters increase, one-handed helices can be obtained with a smaller amount of sergeants. As expected from the above results, a mixed system of **A** and **C_3_(*S*)** exhibited the highest MMP and HRP values ever reported (Fig. S25 and Table S1†) and the large degree of polymerization (Fig. S35†),^10e,i,18b^ as estimated using the amplification model developed by Meijer and coworker.^10*i*^ In contrast to conventional supramolecular helices, our TPA system has no characteristic features, except for the propeller conformation of TPA. Hence, we assume that TPA has an intrinsic potential for an anomalously high degree of chiral amplification in the sergeants and soldiers system. For example, the propeller chirality of TPA is uniquely decided by the handedness of the supramolecular helices. Consequently, handedness inversion requires extra energy to invert the propeller chirality of TPA. Hence, it is reasonable that supramolecular TPA helices exhibit high HRP values.

On the other hand, the MMP primarily originates from the mismatch between the stereogenic centres of the sergeant and the handedness of the supramolecular helices. To reveal the relationship between TPA and the large MMP value, we prepared various sergeants and soldiers based on the molecular designs of **A** and **C_3_(*S*)** (Fig. 1a). All analogues exhibited nearly identical absorption spectra at 25 °C in cyclohexane (Fig. 3a and S21†). Hence, the following discussion is based on the assumption that the TPA derivatives form supramolecular helices with the same conformations as **A** and **C_3_(*S*)**. We investigated the degree of chiral amplification in the mixed systems with **A** and its chiral analogues **C_1m_(*S*)**, **C_1p_(*S*)**, and **C_2m,m_(*S*)**, respectively, whose names indicate the number and positions of the stereogenic centers in their side chains (Fig. S26–28†). As summarized in Fig. 3c, the use of **C_1p_(*S*)** resulted in the lowest chiral amplification, and **C_1m_(*S*)** and **C_2m,m_(*S*)** resulted in nearly identical degrees of chiral amplification to that of **C_3_(*S*)**, suggesting that the role of side-chain chirality is completely different at the *para*- and *meta*-positions. Because side chains at the *meta*-positions sterically hinder the rotation of the phenyl groups to which the side chains are attached, side chains at these positions must affect the propeller conformation of TPA more than those at the *para*-position. Consequently, the MMP of **C_1m_(*S*)** originating from the mismatch between the chiral side chains and the propeller chirality might be larger than that of **C_1p_(*S*)**. An identical trend was observed for soldiers **A_-m_** and **A_-p_**, whose long alkyl chains at the *meta*- and *para*-positions are replaced by methyl groups (Fig. S29 and 30†). When the achiral TPA derivatives were mixed with **C_3_(*S*)**, **A_-p_** resulted in a nearly identical degree of chiral amplification to that of **A**, whereas **A_-m_** exhibited a much lower degree of chiral amplification (Fig. 3d). From the observed unique effects of the side chains at the *meta*-position, we concluded that propeller conformation of TPA enabled effective peripheral packing and realized the large MMP. Interestingly, a mixed system of **A_-p_** and **C_3_(*S*)** had a higher degree of chiral amplification than that of **A** and **C_3_(*S*)** (inset in Fig. 3d); the handedness of the supramolecular helices of **A_-p_** was fully biased by the addition of only 0.2 mol% **C_3_(*S*)**. Because **A_-p_** starts to self-assemble at a higher temperature during the cooling process than **A** and **A_-m_** (Fig. S31†), molecules of **A_-p_** interact better with each other in the resultant helices, potentially underlying the increase in the HRP and MMP values.

**Fig. 4:**
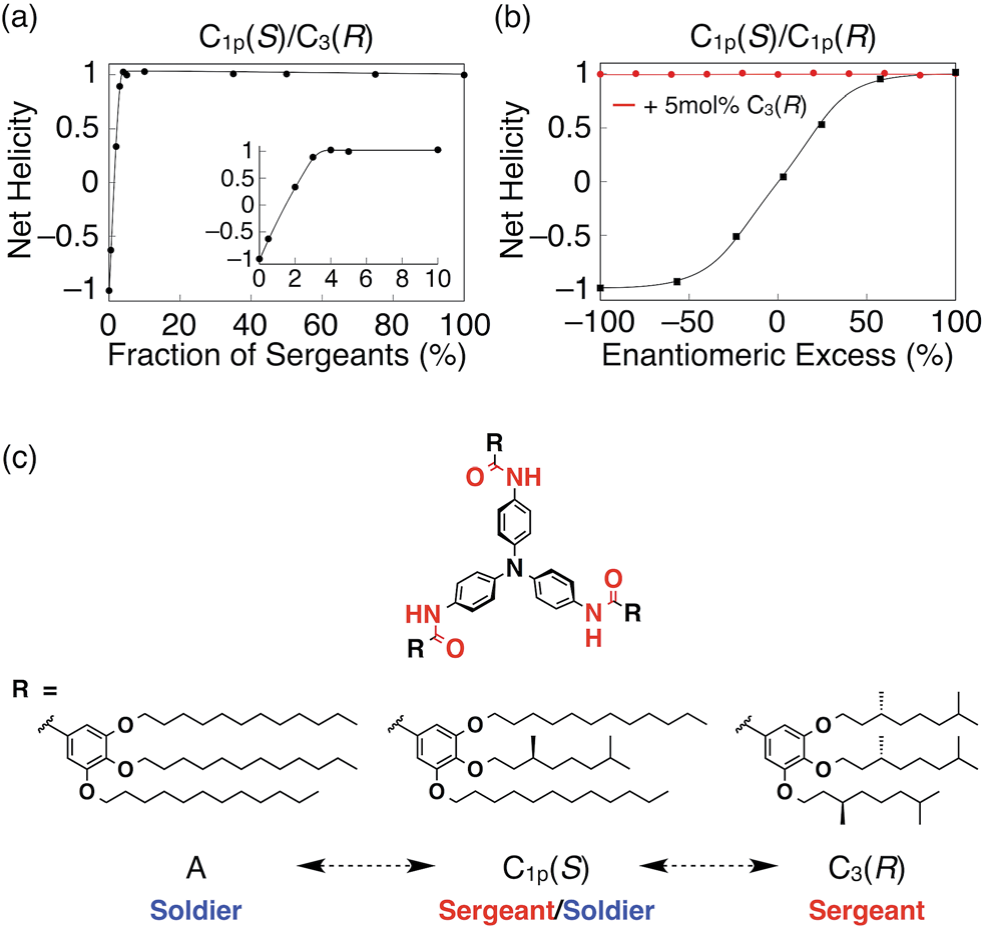
(a) Plot of net helicity against the fraction of sergeants in the mixture of **C_1p_(*S*)**/**C_3_(*R*)**. (b) Plot of net helicity against the enantiomeric excess of **C_1p_(*R*)**/**C_1p_(*S*)** in the presence (red) and absence (black) of 5 mol% **C_3_(*R*)**. The total TPA concentration and the experimental temperatures were 30 μM and 25 °C, respectively, for all measurements. (c) Schematic illustration of the hierarchy in the sergeants and soldiers systems of propeller-shaped TPA derivatives..

The constituents of supramolecular helices are in equilibrium between the monomeric and aggregate (helix) states.^4b,e^ Hence, to obtain a high degree of chiral amplification in these dynamic systems, sergeants and soldiers must co-aggregate efficiently. The MMP of the supramolecular helices increases after introducing larger numbers of chiral side chains per sergeant. However, this approach is incompatible with efficient co-aggregation because the chiral side chains decrease the association constant of the sergeants for soldiers because of steric hindrance.^10m,18b^ However, in the TPA system, because the sergeants and soldiers are both propeller-shaped, the presence of propeller chirality does not lead to the segregation of the sergeants and soldiers, which may be no less important than the MMP and HRP values of TPA systems for chiral amplification. Furthermore, the degree of chiral amplification can be compared over the various TPA derivatives because their helical conformations are always nearly identical, in contrast to supramolecular helices based on planar π-conjugated molecules, such as perylene bisimide.^5*b*^ In addition, it is rare that the degree of chiral amplification drastically changes just by altering the position of the stereogenic centers. By utilizing these unique features, we demonstrated that **C_1p_(*S*)** can switch its role from sergeant to soldier if the partner was changed from **A** to **C_3_(*R*)**. As shown in Fig. 4a, the *g*-value of the mixed system of **C_1p_(*S*)** and **C_3_(*R*)** was saturated with the addition of a 4 mol% fraction of **C_3_(*R*)** (Fig. S32†). By contrast, in the majority rule experiments^10e,19^ for **C_1p_(*R*)**/**C_1p_(*S*)** (Fig. 4b and S33†), the net helicity was constant, independent of the enantiomeric excess of **C_1p_(*R*)** or **C_1p_(*S*)**, if 5 mol% **C_3_(*R*)** was present. Because **C_1p_(*S*)** behaves as a sergeant in the mixed system with **C_1p_(*S*)** and **A** (Fig. 3c and S26†), the observed results suggest hierarchy in the sergeants and soldiers systems of our TPA helices (Fig. 4c). Note that, Meijer and coworkers have reported the similar majority rule experiments using two BTA derivatives with different numbers of stereocenters with opposite stereoconfiguration.^10*j*^ However, in their case, the majority rule was simply dictated by the number of stereocenters, suggesting that propeller conformation of TPA plays an important role for the observed anomalous chiral amplifications.

## Conclusions

In conclusion, we have demonstrated unprecedented chiral amplification of supramolecular helices consisting of TPA derivatives in which the handedness of the helices was fully controlled using a 0.2 mol% fraction of sergeants. Such an unprecedentedly high degree of chiral amplification was achieved *via* the dynamic propeller conformation of TPA. Therefore, this study provides valuable insights into the construction of helical structures and a novel design strategy for supramolecular chiral amplification.
