# Supplementary material for: Dynamic propeller conformation for the unprecedentedly high degree of chiral amplification of supramolecular helices
Source: Chem Sci. 2016 Jul 12;7(11):6689–94. doi: 10.1039/c6sc02814d (PMC5355865; doi:10.1039/c6sc02814d)
Supplement: Supplementary file 1 [file SC-007-C6SC02814D-s001.pdf]

Supporting Information

Dynamic Propeller Chirality for The Unprecedentedly High Degree of Chiral Amplification of Supramolecular Helices

Taehoon Kim, Tadashi Mori, Takuzo Aida and Daigo Miyajima*

E-mail: daigo.miyajima@riken.jp (D.M.)

Table of Contents

1. **General** S2
2. **Synthesis**
   - **2.1** Synthesis of **m, 1m(*S*), 1p(*S*), 1p(*R*),** and **2m,m(*S*)** S3–S5
   - **2.2** Synthesis of **mA, 1mA(*S*), 1pA(*S*), 1pA(*R*)** and **2m,mA(*S*)** S6–S8
   - **2.3** Synthesis of **A**, $\mathbf{A_{-p}}$, $\mathbf{A_{-m}}$, $\mathbf{C_{1m}}$**(*S*)**, $\mathbf{C_{1p}}$**(*S*)**, $\mathbf{C_{1p}}$**(*R*)**, $\mathbf{C_{2m,m}}$**(*S*)**, $\mathbf{C_3}$**(*S*)** and $\mathbf{C_3}$**(*R*)** S8–S14
3. **Analytical Data**
   - **3.1** ^{1}H and ^{13}C NMR Spectroscopy S15–S23
   - **3.2** MALDI-TOF Mass Spectrometry S24–S28
4. **Supplementary Figures**
   - **4.1** DLS and Electronic Absorption Profiles of **A** S29
   - **4.2** AFM Height Image Analysis of Polymerized **A** S30
   - **4.3** Electronic Absorption Spectra of TPA Derivatives S31
   - **4.4** FT-IR spectra of $\mathbf{C_3}$**(*R*)**, $\mathbf{C_3}$**(*S*)** and 9:1 Mixtures of **A/**$\mathbf{C_3}$**(*R*)** and **A/**$\mathbf{C_3}$**(*S*)** S32
   - **4.5** Sergeants and Soldiers Experiments for Mixtures of **A/**$\mathbf{C_3}$**(*S*)** (1) S33
   - **4.6** Sergeants and Soldiers Experiments for Mixtures of **A/**$\mathbf{C_3}$**(*S*)** (2) S34
   - **4.7** Theoretical Estimation of the MMP and HRP values S35
   - **4.8** Sergeants and Soldiers Experiments for Mixtures of **A/**$\mathbf{C_{1p}}$**(*S*)** S36
   - **4.9** Sergeants and Soldiers Experiments for Mixtures of **A/**$\mathbf{C_{1m}}$**(*S*)** S37
   - **4.10** Sergeants and Soldiers Experiments for Mixtures of **A/**$\mathbf{C_{2m,m}}$**(*S*)** S38
   - **4.11** Sergeants and Soldiers Experiments for Mixtures of $\mathbf{A_{-p}}$**/**$\mathbf{C_3}$**(*S*)** S39
   - **4.12** Sergeants and Soldiers Experiments for Mixtures of $\mathbf{A_{-m}}$**/**$\mathbf{C_3}$**(*S*)** S40
   - **4.13** Comparison of the Self-assembly Behavior of **A**, $\mathbf{A_{-p}}$, and $\mathbf{A_{-m}}$ S41
   - **4.14** Sergeants and Soldiers Experiments for Mixtures of $\mathbf{C_{1p}}$**(*S*)/**$\mathbf{C_3}$**(*R*)** S42
   - **4.15** Majority Rule Experiments for Mixtures of $\mathbf{C_{1p}}$**(*R*)/**$\mathbf{C_{1p}}$**(*S*)** (1) S43
   - **4.16** Majority Rule Experiments for Mixtures of $\mathbf{C_{1p}}$**(*R*)/**$\mathbf{C_{1p}}$**(*S*)** (2) S44
   - **4.17** Theoretical Estimation of Number Averaged Degree of Polymerization S45
   - **4.18** Error Ranges of Sergeants and Soldiers Experiments S46
5. **References** S47

1. General

Materials and me Unless otherwise indicated, all starting materials were obtained from commercial suppliers (Wako Pure Chemical, Kanto, and TCI, etc.) and were used without purification. Methylene chloride, hexane, DMF, and Ethanol were distilled before use.

Methods: Visualization of synthesized compounds was accomplished with UV light, iodine vapor or by staining using base solution of cerium ammonium molybdate. Flash chromatography was carried out with Silica Gel 60 (230-400 mesh) from Wako Pure Chemical Industries, Ltd. Recycling preparative high-performance liquid chromatography (HPLC) was performed using a YMC-GPC column on an YMC LC-Forte/R. ^{1}H and ^{13}C NMR spectra were recorded at 25 °C on a JEOL model JNM-ECA500 spectrometer, operating at 500 and 125 MHz, respectively, where chemical shifts (δ in ppm) were determined using tetramethylsilane as an internal reference. Matrix-assisted laser desorption ionization time-of-flight (MALDI–TOF) mass spectrometry was performed in the reflector mode on a Brucker model autoflex TM speed spectrometer. Electronic absorption and circular dichroism (CD) spectra were recorded on a JASCO model V-670 UV/VIS/NIR spectrophotometer and a JASCO Type J-820 spectropolarimeter, respectively, using a quartz cell of 10 mm optical path length. Dynamic light scattering (DLS) measurements were performed with a Malvern type Zetasizer Nano ZSP at 25 °C using a quartz cell of 10 mm optical path length. Infrared spectra were recorded using a JASCO model FT/IR-610Plus Fourier transform infrared spectrometer. Vibrational circular dichroism (VCD) spectra were measured in a 0.5 mm BaF_2 cell with JASCO FVS-6000. All VCD spectra were collected for ca. 2-3 h at a resolution of 4 cm^{-1}. g-values used in this manuscript is calculated from the following equation.

$$g = \frac{\Delta\varepsilon}{\varepsilon} = \frac{A^l - A^r}{A} = \frac{CD - effect\ [mdeg]}{32982 \cdot A}$$

where ε is molar ellipticity, A represents the conventional absorbance of nonpolarized light, A^l and A^r are the absorptions of left and right circularly polarized light, respectively.[S1]

2. Synthesis

Synthesis of 3,4-bis(dodecyloxy)-5-hydroxy methyl benzoate, **1m'**,[S2] 4-[(*S*)-3,7-dimethyloctyl oxy]-3,5-dihydroxy methyl benzoate, **1p'(*S*)**,[S2] 4-[(*R*)-3,7-dimethyloctyloxy]-3,5-dihydroxy methyl benzoate, **1p'(*R*)**,[S2] 4-dodecyloxy-3,5-dihydroxy methyl benzoate, **2m,m'**,[S3] 3,5-bis (dodecyloxy)-4-methoxybenzoic acid, **pA**,[S4] 3,4,5-tris(dodecyloxy)benzoic acid, **A'**,[S2] 3,4,5 -[tris-(*S*)-3,7-dimethyloctyloxy]benzoic acid, **3A(*S*)**[S5] and 3,4,5-[tris-(*R*)-3,7-dimethyloctyl oxy]benzoic acid, **3A(*R*)**,[S5] were described previously. The other substituted 3,4,5-trisalkoxy benzoic acid were synthesized by means of similar procedures. (*R*)-3,7-dimethyloctyl bromide[S6] and (*S*)-3,7-dimethyloctyl bromide[S7] was synthesized from (*R*)-citronellyl bromide and (*S*)-citronellol, respectively, as previous report.

2.1 Synthesis of m, 1m(*S*), 1p(*S*), 1p(*R*), and 2m,m(*S*)

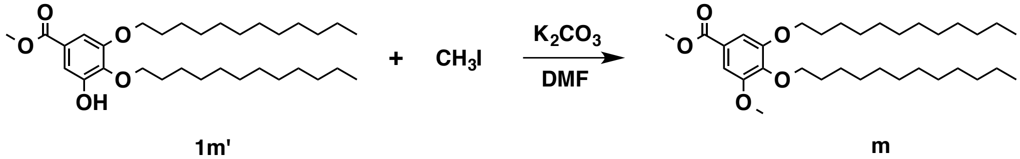

1m' m

m: To a DMF solution (20 mL) of a mixture of **1m'** (150 mg, 0.29 mmol) and excess K_2CO_3 was added iodomethane (46 mg, 0.33 mmol), and the mixture was stirred for 12 h at 80 °C under a positive pressure of argon. The reaction mixture was washed twice with water (50 mL), dried over Na_2SO_4, and evaporated to dryness under reduced pressure. The residue was chromatographed on silica gel with hexane/AcOEt (9/1 v/v) as an eluent, where the first fraction was collected and evaporated under reduced pressure to give **m** as colorless liquid (140 mg, 0.25 mmol, 86%). ^{1}H NMR (500 MHz, $CDCl_3$, 25 °C, TMS) δ (ppm) = 7.27 (s, 2H), 4.02 (q, *J* = 6.3 Hz, 4H), 3.89 (s, 3H), 3.88 (s, 3H), 1.82 (quint, *J* = 6.9 Hz, 2H), 1.75 (quint, *J* = 6.9 Hz, 2H), 1.53-1.09 (overlapped m, 36H), 0.88 (t, *J* = 6.9 Hz, 6H); ^{13}C NMR (125 MHz, $CDCl_3$, 25 °C, TMS) δ (ppm) = 166.8, 152.9, 141.8, 124.7, 107.9, 106.5, 73.5, 69.0, 56.2, 52.2, 31.9, 30.3-25.9, 22.6, 14.1.

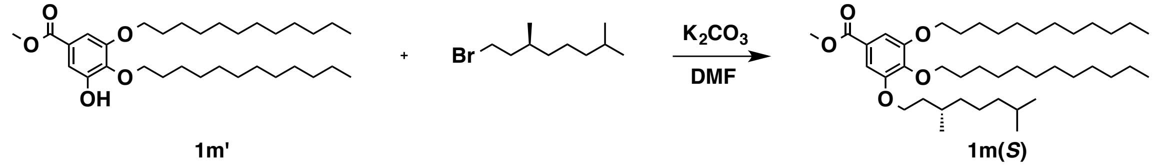

1m' 1m(*S*)

1m(*S*): To a DMF solution (20 mL) of a mixture of **1m'** (100 mg, 0.19 mmol) and excess K_2CO_3 was added (*S*)-3,7-dimethyloctyl bromide (46 mg, 0.21 mmol), and the mixture was stirred for 12 h at 80 °C under a positive pressure of argon. The reaction mixture was washed twice with water (50 mL), dried over Na_2SO_4, and evaporated to dryness under reduced pressure. The residue was chromatographed on silica gel with hexane/AcOEt (9/1 v/v) as an eluent, where the first fraction was collected and evaporated under reduced pressure to give **1m(*S*)** as colorless liquid (110 mg, 0.17 mmol, 89%). ^1H NMR (500 MHz, $CDCl_3$, 25 °C, TMS) δ (ppm) = 7.25 (overlapped s, J = 2.3 Hz, 2H), 4.06-3.9 (m, 6H), 3.89 (s, 3H), 1.82-1.66 (m, 6H), 1.51-1.12 (overlapped m, 44H), 0.94 (d, *J* = 6.9 Hz, 3H),0.88 (m, 12H); ^{13}C NMR (125 MHz, $CDCl_3$, 25 °C, TMS) δ (ppm) = 166.9, 152.8, 142.3, 124.6, 107.9, 73.5, 69.1, 67.4, 52.1, 39.2, 37.3, 36.3, 31.9, 30.3, 29.8-26.0, 24.7, 22.6, 19.6, 14.1.

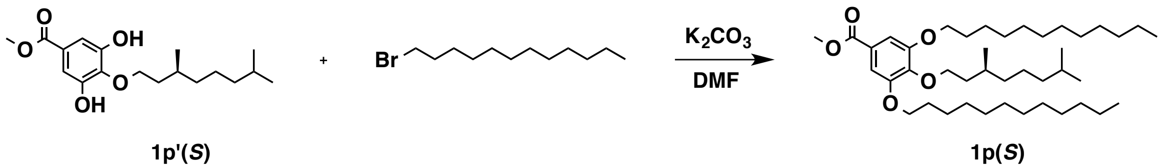

1p(*S*): To a DMF solution (50 mL) of a mixture of **1p'(*S*)** (300 mg, 0.92 mmol) and excess K_2CO_3 was added 1-bromododecane (691 mg, 2.78 mmol), and the mixture was stirred for 12 h at 80 °C under a positive pressure of argon. The reaction mixture was washed twice with water (100 mL), dried over Na_2SO_4, and evaporated to dryness under reduced pressure. The residue was chromatographed on silica gel with hexane/AcOEt (9/1 v/v) as an eluent, where the first fraction was collected and evaporated under reduced pressure to give **1p(*S*)** as colorless liquid (525 mg, 0.78 mmol, 85%). ^1H NMR (500 MHz, $CDCl_3$, 25 °C, TMS) δ (ppm) = 7.25 (s, 2H), 4.18-3.90 (m, 6H), 3.89 (s, 3H), 1.86-1.68 (m, 6H), 1.57-1.44 (m, 6H), 1.40-1.09 (overlapped m, 42H), 0.92 (d, *J* = 6.9 Hz, 3H),0.87 (m, 12H); ^{13}C NMR (125 MHz, $CDCl_3$, 25 °C, TMS) δ (ppm) = 166.9, 152.8, 142.4, 124.6, 107.9, 71.7, 69.1, 39.3, 37.5, 31.9, 29.7-26.1, 24.7, 22.6, 19.5, 14.1.

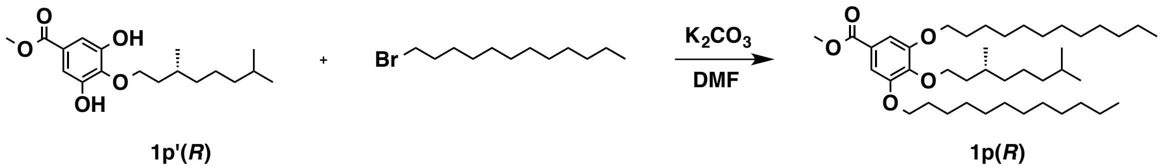

1p(*R*): To a DMF solution (30 mL) of a mixture of **1p'(*R*)** (200 mg, 0.62 mmol) and excess K_2CO_3 was added 1-bromododecane (338 mg, 1.36 mmol), and the mixture was stirred for 12 h at 80 °C under a positive pressure of argon. The reaction mixture was washed twice with water (60 mL), dried over Na_2SO_4, and evaporated to dryness under reduced pressure. The residue was chromatographed on silica gel with hexane/AcOEt (9/1 v/v) as an eluent, where the first fraction was collected and evaporated under reduced pressure to give **1p(*R*)** as colorless liquid (330 mg, 0.50 mmol, 81%). ^{1}H NMR (500 MHz, $CDCl_3$, 25 °C, TMS) δ (ppm) = 7.25 (s, 2H), 4.18-3.90 (m, 6H), 3.89 (s, 3H), 1.86-1.68 (m, 6H), 1.57-1.44 (m, 6H), 1.40-1.09 (overlapped m, 42H), 0.92 (d, J = 6.9 Hz, 3H), 0.87 (m, 12H); ^{13}C NMR (125 MHz, $CDCl_3$, 25 °C, TMS) δ (ppm) = 166.9, 152.8, 142.4, 124.6, 107.9, 71.7, 69.1, 39.3, 37.5, 31.9, 29.7-26.1, 24.7, 22.6, 19.5, 14.1.

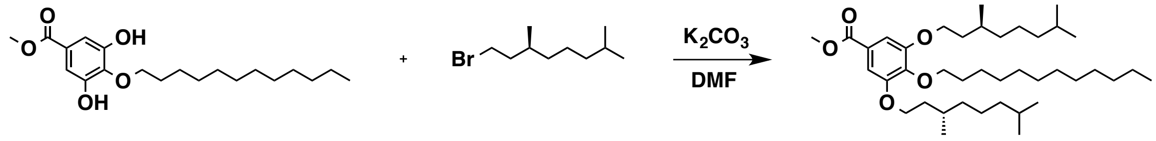

2m,m' 2m,m(*S*)

2m,m(*S*): To a DMF solution (50 mL) of a mixture of **2m,m'** (300 mg, 0.92 mmol) and excess K_2CO_3 was added (*S*)-3,7-dimethyloctyl bromide (472 mg, 2.78 mmol), and the mixture was stirred for 12 h at 80 °C under a positive pressure of argon. The reaction mixture was washed twice with water (100 mL), dried over Na_2SO_4, and evaporated to dryness under reduced pressure. The residue was chromatographed on silica gel with hexane/AcOEt (9/1 v/v) as an eluent, where the first fraction was collected and evaporated under reduced pressure to give **2m,m(*S*)** as colorless liquid (475 mg, 0.80 mmol, 87%). ^{1}H NMR (500 MHz, $CDCl_3$, 25 °C, TMS) δ (ppm) = 7.26 (s, 2H), 4.08-3.98 (m, 6H), 3.89 (s, 3H), 1.86 (m, 2H), 1.77-1.42 (m, 10H), 1.39-1.20 (overlapped m, 28H), 0.94 (d, J = 6.9 Hz, 6H), 0.88 (m, 15H); ^{13}C NMR (125 MHz, $CDCl_3$, 25 °C, TMS) δ (ppm) = 166.9, 152.8, 142.3, 124.6, 107.9, 73.4, 67.4, 60.4, 52.1, 39.2, 37.3, 36.3, 31.9, 30.3, 29.8-26.0, 24.7, 22.6, 19.6, 14.1.

2.2 Synthesis of mA, 1mA(*S*), 1pA(*S*), 1pA(*R*) and 2m,mA(*S*)

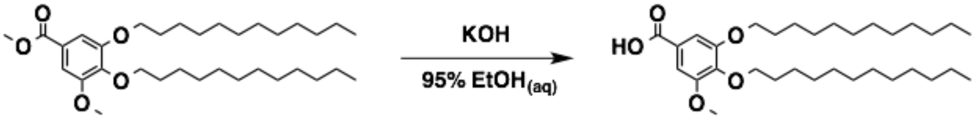

m　　　　mA

mA: To a 95% ethanol solution (20 mL) of a mixture of **m** (140 mg, 0.26 mmol) and KOH (100 mg, 1.79 mmol) was stirred for 2 h at 100 °C under a positive pressure of argon. The emulsion became a clear solution, indicating the progress of the reaction. The reaction mixture was cooled to RT and the solution was acidified with dilute HCl to pH 1. The solution was poured into water (100 mL) to precipitate of a white solid. The residue was chromatographed on silica gel with hexane/AcOEt (3/1 v/v) as an eluent, where the last fraction was collected and evaporated under reduced pressure to give **mA** as white solid (100 mg, 0.19 mmol, 74%). ^{1}H NMR (500 MHz, $CDCl_3$, 25 °C, TMS) δ (ppm) = 7.35 (overlapped s, *J* = 2.9 Hz, 2H), 4.04 (m, 4H), 3.89 (s, 3H), 1.88-1.73 (m, 4H), 1.53-1.21 (overlapped m, 36H), 0.88 (t, *J* = 6.9 and 7.4 Hz, 6H); ^{13}C NMR (125 MHz, $CDCl_3$, 25 °C, TMS) δ (ppm) = 171.3, 153.0, 142.8, 123.6, 107.8, 73.6, 69.2, 56.2, 31.9, 30.2-26.1, 22.7, 14.1.

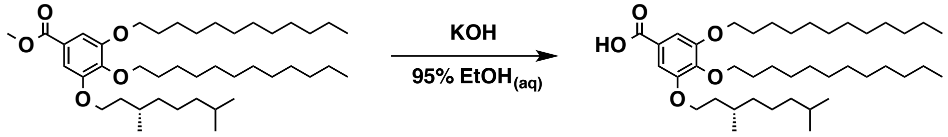

1m(*S*)　　　　1mA(*S*)

1mA(*S*): To a 95% ethanol solution (20 mL) of a mixture of **1m(*S*)** (110 mg, 0.17 mmol) and KOH (65 mg, 1.16 mmol) was stirred for 2 h at 100 °C under a positive pressure of argon. The emulsion became a clear solution, indicating the progress of the reaction. The reaction mixture was cooled to RT and the solution was acidified with dilute HCl to pH 1. The solution was poured into water (100 mL) to precipitate of a white solid. The residue was chromatographed on silica gel with hexane/AcOEt (3/1 v/v) as an eluent, where the last fraction was collected and evaporated under reduced pressure to give **1mA(*S*)** as white solid (100 mg, 0.15 mmol, 88%). ^{1}H NMR (500 MHz, $CDCl_3$, 25 °C, TMS) δ (ppm) = 7.34 (overlapped s, J = 2.9 Hz, 2H), 4.11-3.99 (m, 6H), 1.82-1.12 (overlapped m, 50H), 0.95 (d, *J* = 6.3 Hz, 3H),0.88 (m, 12H); ^{13}C NMR (125 MHz, $CDCl_3$, 25 °C, TMS) δ (ppm) = 172.1, 152.8, 143.1, 123.6, 108.5, 73.5, 69.1, 67.5, 52.1, 39.2, 37.3, 36.2, 31.9, 30.3, 29.8-26.0, 24.7, 22.6, 19.6, 14.1.

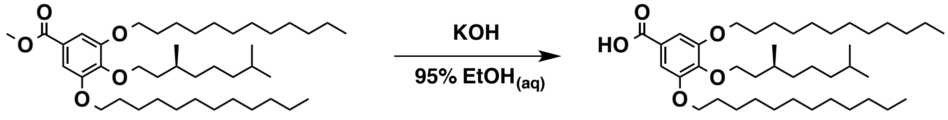

1p(*S*) 1pA(*S*)

1pA(*S*): To a 95% ethanol solution (50 mL) of a mixture of **1p(*S*)** (525 mg, 0.79 mmol) and KOH (312 mg, 5.56 mmol) was stirred for 2 h at 100 °C under a positive pressure of argon. The emulsion became a clear solution, indicating the progress of the reaction. The reaction mixture was cooled to RT and the solution was acidified with dilute HCl to pH 1. The solution was poured into water (200 mL) to precipitate of a white solid. The residue was chromatographed on silica gel with hexane/AcOEt (3/1 v/v) as an eluent, where the last fraction was collected and evaporated under reduced pressure to give **1pA(*S*)** as white solid (450 mg, 0.69 mmol, 87%). ^{1}H NMR (500 MHz, $CDCl_3$, 25 °C, TMS) δ (ppm) = 7.32 (s, 2H), 4.18-3.98 (m, 6H), 1.87-1.67 (m, 6H), 1.57-1.44 (m, 6H), 1.40-1.09 (overlapped m, 42H), 0.93 (d, *J* = 6.3 Hz, 3H),0.87 (m, 12H); ^{13}C NMR (125 MHz, $CDCl_3$, 25 °C, TMS) δ (ppm) = 172.2, 152.9, 143.0, 123.6, 108.5, 71.8, 69.4, 52.1, 39.3, 37.4, 31.9, 29.3-26.1, 24.7, 22.6, 19.5, 14.1.

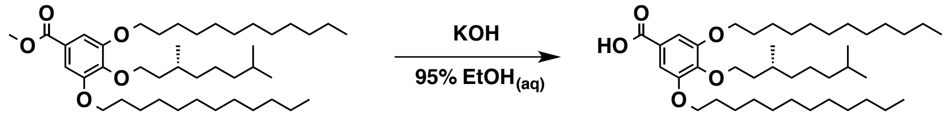

1p(*R*) 1pA(*R*)

1pA(*R*): To a 95% ethanol solution (30 mL) of a mixture of **1p(*R*)** (330 mg, 0.50 mmol) and KOH (196 mg, 3.50 mmol) was stirred for 2 h at 100 °C under a positive pressure of argon. The emulsion became a clear solution, indicating the progress of the reaction. The reaction mixture was cooled to RT and the solution was acidified with dilute HCl to pH 1. The solution was poured into water (150 mL) to precipitate of a white solid. The residue was chromatographed on silica gel with hexane/AcOEt (3/1 v/v) as an eluent, where the last fraction was collected and evaporated under reduced pressure to give **1pA(*R*)** as white solid (324 mg, 0.49 mmol, 99%). ^{1}H NMR (500 MHz, $CDCl_3$, 25 °C, TMS) δ (ppm) = 7.32 (s, 2H), 4.18-3.98 (m, 6H), 1.87-1.67 (m, 6H), 1.57-1.44 (m, 6H), 1.40-1.09 (overlapped m, 42H), 0.93 (d, *J* = 6.3 Hz, 3H),0.87 (m, 12H); ^{13}C NMR (125 MHz, $CDCl_3$, 25 °C, TMS) δ (ppm) = 172.2, 152.9, 143.0, 123.6, 108.5, 71.8, 69.4, 52.1, 39.3, 37.4, 31.9, 29.3-26.1, 24.7, 22.6, 19.5, 14.1.

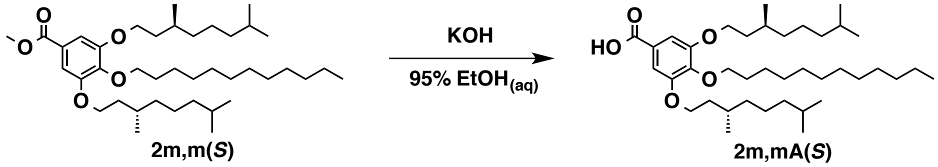

2m,mA(*S*): To a 95% ethanol solution (50 mL) of a mixture of **2m,m(*S*)** (475 mg, 0.75 mmol) and KOH (295 mg, 5.26 mmol) was stirred for 2 h at 100 °C under a positive pressure of argon. The emulsion became a clear solution, indicating the progress of the reaction. The reaction mixture was cooled to RT and the solution was acidified with dilute HCl to pH 1. The solution was poured into water (200 mL) to precipitate of a white solid. The residue was chromatographed on silica gel with hexane/AcOEt (3/1 v/v) as an eluent, where the last fraction was collected and evaporated under reduced pressure to give **2m,mA(*S*)** as white solid (420 mg, 0.67 mmol, 90%). ^{1}H NMR (500 MHz, $CDCl_3$, 25 °C, TMS) δ (ppm) = 7.33 (s, 2H), 4.11-4.01 (m, 6H), 1.87 (m, 2H), 1.78-1.43 (m, 10H), 1.39-1.20 (overlapped m, 28H), 0.95 (d, *J* = 6.9 Hz, 6H),0.88 (m, 15H); ^{13}C NMR (125 MHz, $CDCl_3$, 25 °C, TMS) δ (ppm) = 171.5, 152.8, 143.1, 123.6, 108.5, 73.5, 67.5, 65.0, 58.5, 39.2, 37.3, 36.2, 31.9, 30.3, 29.8-26.0, 24.7, 22.6, 19.6, 14.1.

2.3 Synthesis of A, A_{-p}, A_{-m}, C_{1m}(*S*), C_{1p}(*S*), C_{1p}(*R*), $C_{2m,m}$(*S*), C_3(*S*) and C_3(*R*)

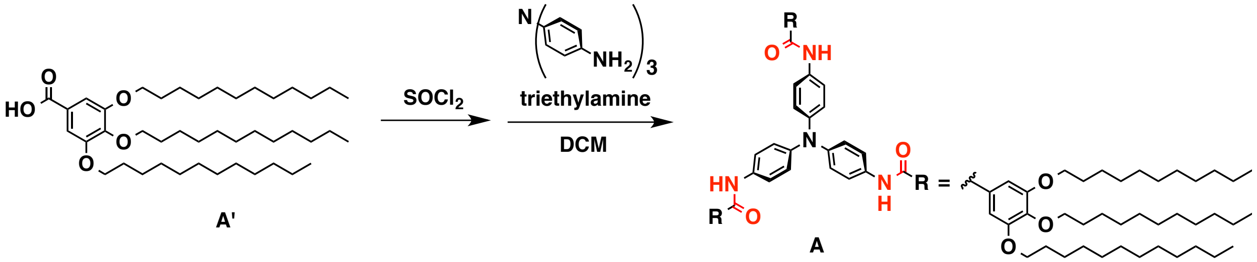

A: A thionyl chloride (50 mL) solution of **A’** (1.02 g, 1.48 mmol) was refluxed for 6 h, and the reaction mixture was evaporated to dryness. To a N-Methyl-2-pyrrolidone (NMP) solution (50 mL) of the residue were successively added a mixture of tris(4-aminophenyl) amine (100 mg, 0.37 mmol), pyridine (5 mL) and excess LiCl was stirred for 2 h at 80 °C under a positive pressure of argon. The solution was poured into ethanol (200 mL) to precipitate of a white solid. The residue was chromatographed on silica gel with $CHCl_3$/EtOH (99/1 v/v) as an eluent, where the second fraction was collected and evaporated to dryness. The residue was subjected to recycling preparative HPLC (YMC LC-forte/R) with $CHCl_3$ as an eluent at a flow rate of 10 mL min^{-1}, where the first fraction was collected and evaporated to dryness under reduced

pressure to give **A** as white solid (2.24 g, 0.25 mmol, 68%). ^{1}H NMR (500 MHz, $CDCl_3$, 25 °C, TMS) δ (ppm) = 7.83 (s, 3H), 7.50 (d, *J* = 9.2 Hz, 6H), 7.06 (m, 12H), 4.00 (m, 12H), 1.83-1.72 (m, 18H), 1.52-1.15 (overlapped m, 168H), 0.88 (m, 27H); ^{13}C NMR (125 MHz, $CDCl_3$, 25 °C, TMS) δ (ppm) = 165.6, 153.2, 144.2, 141.5, 132.9, 129.8, 124.4, 121.6, 105.8, 73.6, 69.4, 31.9, 30.3-29.3, 26.1, 22.7, 14.1. MALDI-TOF mass: m/z calculated, for compound **A** $[M + H]^+$, 2259.88; Found: $[M + H]^+$, 2260.16.

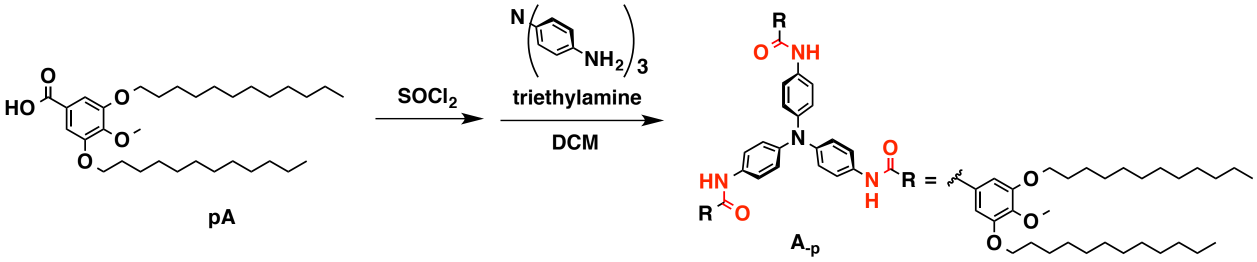

A$_{-p}$: A thionyl chloride (10 mL) solution of **pA** (223 mg, 0.43 mmol) was refluxed for 6 h, and the reaction mixture was evaporated to dryness. To a NMP solution (20 mL) of the residue were successively added a mixture of tris(4-aminophenyl) amine (31 mg, 0.11 mmol), pyridine (2 mL) and excess LiCl was stirred for 2 h at 80 °C under a positive pressure of argon. The solution was poured into ethanol (100 mL) to precipitate of a white solid. The residue was chromatographed on silica gel with $CHCl_3$/EtOH (99/1 v/v) as an eluent, where the second fraction was collected and evaporated to dryness. The residue was subjected to recycling preparative HPLC (YMC LC-forte/R) with $CHCl_3$ as an eluent at a flow rate of 10 mL min^{-1}, where the first fraction was collected and evaporated to dryness under reduced pressure to give **A$_{-p}$** as white solid (122 mg, 0.27 mmol, 63%). ^{1}H NMR (500 MHz, $CDCl_3$, 25 °C, TMS) δ (ppm) = 7.74 (s, 3H), 7.51 (d, *J* = 9.2 Hz, 6H), 7.08 (d, *J* = 9.2 Hz, 6H), 7.05 (s, 6H), 4.04 (t, *J* = 6.3 Hz, 12H), 3.89 (s, 9H), 1.82 (quint, *J* = 6.9 Hz, 12H), 1.51-1.21 (overlapped m, 108H), 0.88 (t, *J* = 6.9 Hz, 18H); ^{13}C NMR (125 MHz, $CDCl_3$, 25 °C, TMS) δ (ppm) = 165.5, 153.0, 144.2, 142.1, 132.9, 130.0, 124.4, 121.5, 105.8, 69.5, 60.8, 31.9, 29.7-29.3, 26.0, 22.7, 14.1. MALDI-TOF mass: m/z calculated, for compound **A$_{-p}$** $[M + H]^+$, 1797.36; Found: $[M + H]^+$, 1797.60.

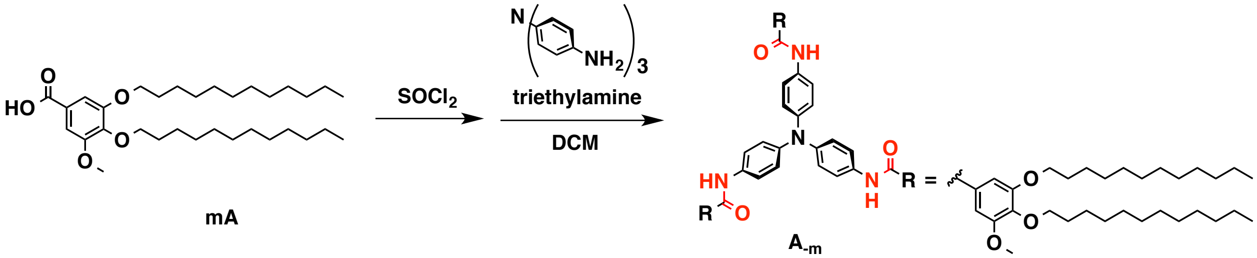

A$_{\text{-m}}$: A thionyl chloride (5 mL) solution of **mA** (100 mg, 0.19 mmol) was refluxed for 6 h, and the reaction mixture was evaporated to dryness. To a NMP solution (10 mL) of the residue were successively added a mixture of tris(4-aminophenyl) amine (16 mg, 0.054 mmol), pyridine (1 mL) and excess LiCl was stirred for 2 h at 80 °C under a positive pressure of argon. The solution was poured into ethanol (50 mL) to precipitate of a white solid. The residue was chromatographed on silica gel with $CHCl_3$/EtOH (99/1 v/v) as an eluent, where the second fraction was collected and evaporated to dryness. The residue was subjected to recycling preparative HPLC (YMC LC-forte/R) with $CHCl_3$ as an eluent at a flow rate of 10 mL min^{-1}, where the first fraction was collected and evaporated to dryness under reduced pressure to give **A$_{\text{-m}}$** as white solid (67 mg, 0.037 mmol, 69%). ^{1}H NMR (500 MHz, $CDCl_3$, 25 °C, TMS) δ (ppm) = 7.86 (s, 3H), 7.45 (d, J = 9.2 Hz, 6H), 7.06 (m, 12H), 4.01 (m, 12H), 3.87 (s, 9H), 1.86-1.71 (m, 12H), 1.51-1.21 (overlapped m, 108H), 0.88 (m, 18H); ^{13}C NMR (125 MHz, $CDCl_3$, 25 °C, TMS) δ (ppm) = 165.5, 153.3, 144.2, 141.0, 132.8, 129.8, 124.3, 121.6, 105.7, 104.4, 73.6, 69.3, 56.4, 31.9, 30.2-29.4, 26.0, 22.7, 14.1. MALDI-TOF mass: m/z calculated, for compound **A$_{\text{-m}}$** $[M + H]^+$, 1797.36; Found: $[M + H]^+$, 1797.58.

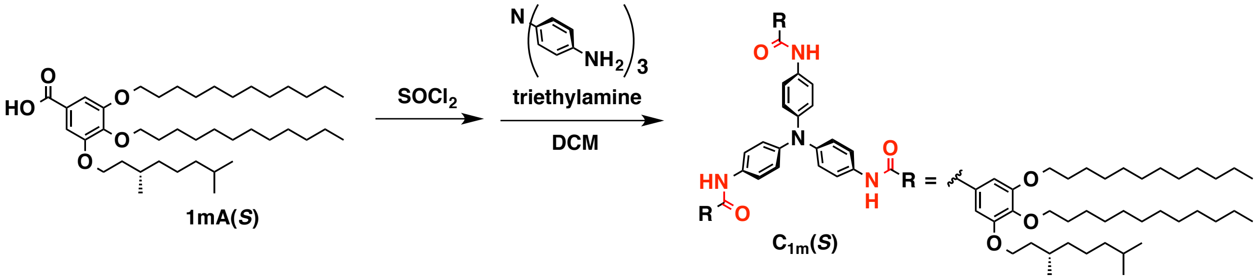

C$_{\text{1m}}$(*S*): A thionyl chloride (5 mL) solution of **1mA(*S*)** (100 mg, 0.15 mmol) was refluxed for 6 h, and the reaction mixture was evaporated to dryness. To a NMP solution (10 mL) of the residue were successively added a mixture of tris(4-aminophenyl) amine (11 mg, 0.039 mmol), pyridine (1 mL) and excess LiCl was stirred for 2 h at 80 °C under a positive pressure of argon.

The solution was poured into ethanol (50 mL) to precipitate of a white solid. The residue was chromatographed on silica gel with $CHCl_3$/EtOH (99/1 v/v) as an eluent, where the second fraction was collected and evaporated to dryness. The residue was subjected to recycling preparative HPLC (YMC LC-forte/R) with $CHCl_3$ as an eluent at a flow rate of 10 mL min^{-1}, where the first fraction was collected and evaporated to dryness under reduced pressure to give **C_{1m}(*S*)** as white solid (57 mg, 0.026 mmol, 67%). ^{1}H NMR (500 MHz, $CDCl_3$, 25 °C, TMS) δ (ppm) = 7.67 (s, 3H), 7.51 (d, *J* = 8.6 Hz, 6H), 7.09 (d, *J* = 8.6 Hz, 6H), 7.04 (ss, 6H), 4.03 (m, 18H), 1.92-1.10 (overlapped m, 150H), 0.95 (d, J = 6.9 Hz, 9H), 0.88 (m, 36H); ^{13}C NMR (125 MHz, $CDCl_3$, 25 °C, TMS) δ (ppm) = 165.5, 153.3, 141.5, 132.9, 129.9, 124.4, 121.4, 105.7, 73.5, 67.8, 39.2, 37.3, 36.4, 31.9, 30.3-29.4, 28.0, 26.1, 24.7, 22.6, 19.6, 14.1. MALDI-TOF mass: m/z calculated, for compound **C_{1m}(*S*)** $[M + H]^+$, 2175.78; Found: $[M + H]^+$, 2176.12.

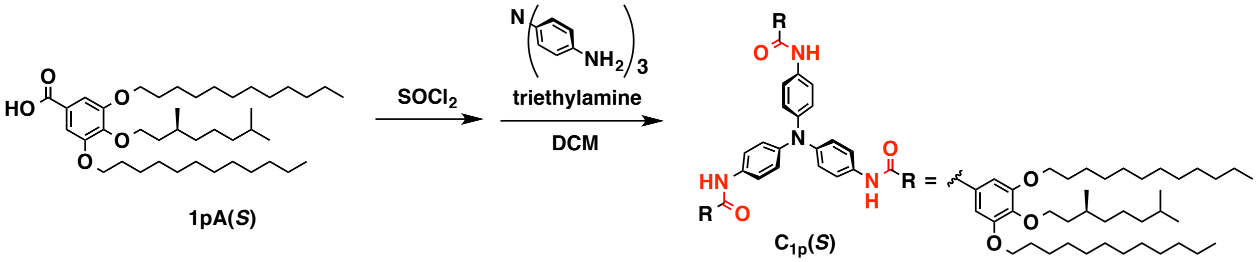

C_{1p}(S): A thionyl chloride (10 mL) solution of **1pA(*S*)** (220 mg, 0.34 mmol) was refluxed for 6 h, and the reaction mixture was evaporated to dryness. To a NMP solution (20 mL) of the residue were successively added a mixture of tris(4-aminophenyl) amine (25 mg, 0.085 mmol), pyridine (2 mL) and excess LiCl was stirred for 2 h at 80 °C under a positive pressure of argon. The solution was poured into ethanol (100 mL) to precipitate of a white solid. The residue was chromatographed on silica gel with $CHCl_3$/EtOH (99/1 v/v) as an eluent, where the second fraction was collected and evaporated to dryness. The residue was subjected to recycling preparative HPLC (YMC LC-forte/R) with $CHCl_3$ as an eluent at a flow rate of 10 mL min^{-1}, where the first fraction was collected and evaporated to dryness under reduced pressure to give **C_{1p}(*S*)** as white solid (67 mg, 0.031 mmol, 36%). ^{1}H NMR (500 MHz, $CDCl_3$, 25 °C, TMS) δ (ppm) = 7.81 (s, 3H), 7.50 (d, *J* = 8.6 Hz, 26H), 7.06 (m, 12H), 4.04 (m, 18H), 1.92-1.67 (m, 18H), 1.58-1.42 (m, 18H), 1.40-1.12 (overlapped m, 114H), 0.93 (d, J = 6.3 Hz, 9H), 0.88 (m, 36H); ^{13}C NMR (125 MHz, $CDCl_3$, 25 °C, TMS) δ (ppm) = 165.5, 153.2, 144.1, 141.4, 132.9, 129.8, 124.3, 121.5, 105.7, 71.8, 69.4, 39.3, 37.4, 31.9, 29.7-29.4, 28.0, 26.1, 24.7, 22.6, 19.5,

14.1. MALDI-TOF mass: m/z calculated, for compound $\mathbf{C_{1p}}$(***S***) [M + H]$^+$, 2175.78; Found: [M + H]$^+$, 2176.01.

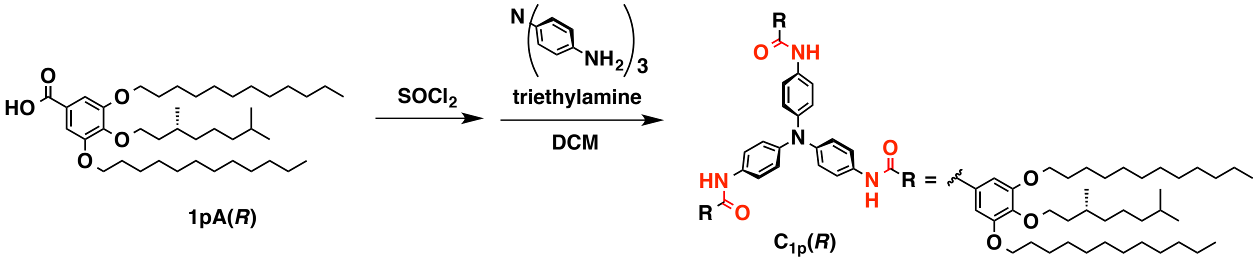

$\mathbf{C_{1p}}$(***R***): A thionyl chloride (10 mL) solution of **1pA(*R*)** (332 mg, 0.50 mmol) was refluxed for 6 h, and the reaction mixture was evaporated to dryness. To a NMP solution (20 mL) of the residue were successively added a mixture of tris(4-aminophenyl) amine (36 mg, 0.13 mmol), pyridine (2 mL) and excess LiCl was stirred for 2 h at 80 °C under a positive pressure of argon. The solution was poured into ethanol (100 mL) to precipitate of a white solid. The residue was chromatographed on silica gel with $CHCl_3$/EtOH (99/1 v/v) as an eluent, where the second fraction was collected and evaporated to dryness. The residue was subjected to recycling preparative HPLC (YMC LC-forte/R) with $CHCl_3$ as an eluent at a flow rate of 10 mL min^{-1}, where the first fraction was collected and evaporated to dryness under reduced pressure to give $\mathbf{C_{1p}}$(***R***) as white solid (95 mg, 0.044 mmol, 34%). ^{1}H NMR (500 MHz, $CDCl_3$, 25 °C, TMS) δ (ppm) = 7.69 (s, 3H), 7.50 (d, *J* = 8.6 Hz, 26H), 7.07 (m, 12H), 4.05 (m, 18H), 1.90-1.67 (m, 18H), 1.58-1.43 (m, 18H), 1.41-1.12 (overlapped m, 114H), 0.93 (d, J = 6.3 Hz, 9H), 0.88 (m, 36H); ^{13}C NMR (125 MHz, $CDCl_3$, 25 °C, TMS) δ (ppm) = 165.5, 153.3, 144.2, 141.5, 133.0, 129.9, 124.4, 121.5, 105.8, 71.8, 69.5, 39.4, 37.5, 32.0, 29.7-29.4, 28.0, 26.1, 24.7, 22.7, 19.6, 14.1. MALDI-TOF mass: m/z calculated, for compound $\mathbf{C_{1p}}$(***R***) [M + H]$^+$, 2175.78; Found: [M + H]$^+$, 2175.79.

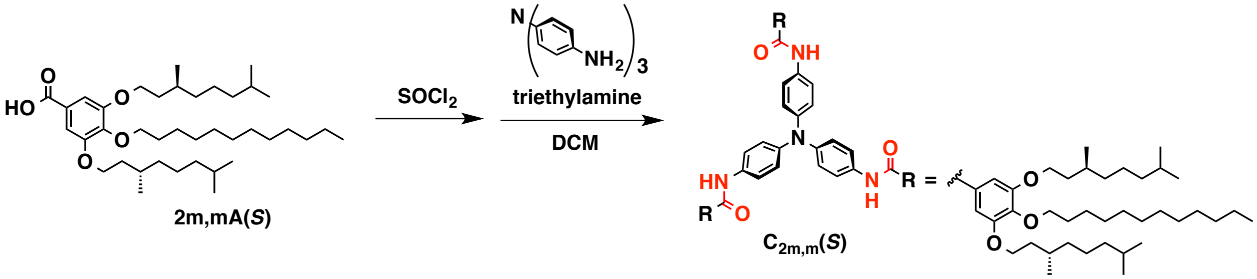

$C_{2m,m}$(*S*): A thionyl chloride (10 mL) solution of **2m,mA(*S*)** (210 mg, 0.34 mmol) was refluxed for 6 h, and the reaction mixture was evaporated to dryness. To a NMP solution (20 mL) of the residue were successively added a mixture of tris(4-aminophenyl) amine (25 mg, 0.085 mmol), pyridine (2 mL) and excess LiCl was stirred for 2 h at 80 °C under a positive pressure of argon. The solution was poured into ethanol (100 mL) to precipitate of a white solid. The residue was chromatographed on silica gel with $CHCl_3$/EtOH (99/1 v/v) as an eluent, where the second fraction was collected and evaporated to dryness. The residue was subjected to recycling preparative HPLC (YMC LC-forte/R) with $CHCl_3$ as an eluent at a flow rate of 10 mL min^{-1}, where the first fraction was collected and evaporated to dryness under reduced pressure to give **$C_{2m,m}$(*S*)** as white solid (98 mg, 0.048 mmol, 56%). ^{1}H NMR (500 MHz, $CDCl_3$, 25 °C, TMS) δ (ppm) = 7.86 (s, 3H), 7.50 (d, *J* = 9.2 Hz, 6H), 7.06 (m, 12H), 4.02 (m, 18H), 1.91-1.43 (m, 36H), 1.41-1.08 (overlapped m, 74H), 0.93 (d, J = 6.9 Hz, 18H), 0.87 (m, 45H); ^{13}C NMR (125 MHz, $CDCl_3$, 25 °C, TMS) δ (ppm) = 165.6, 153.2, 144.1, 141.4, 132.9, 129.8, 124.3, 121.6, 105.7, 73.5, 67.7, 39.2, 37.3, 36.3, 31.9, 30.3-29.4, 28.0, 26.1, 24.7, 22.6, 19.6, 14.1. MALDI-TOF mass: m/z calculated, for compound **$C_{2m,m}$(*S*)** $[M + H]^+$, 2091.69; Found: $[M + H]^+$, 2091.97.

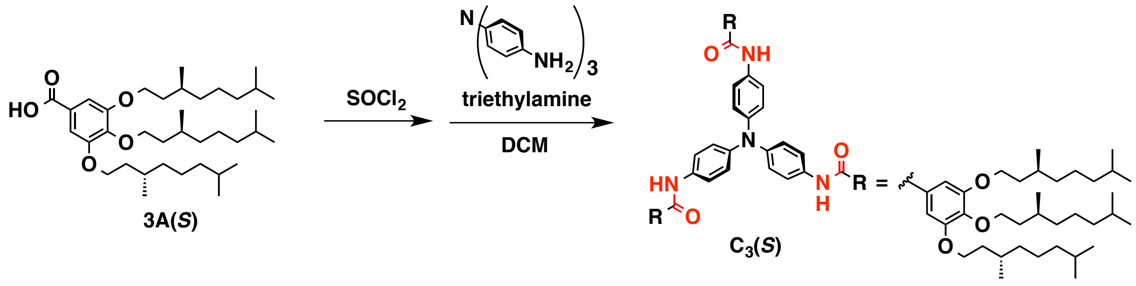

C_3(*S*): A thionyl chloride (30 mL) solution of **3A(*S*)** (481 mg, 0.79 mmol) was refluxed for 6 h, and the reaction mixture was evaporated to dryness. To a NMP solution (30 mL) of the residue were successively added a mixture of tris(4-aminophenyl) amine (65 mg, 0.22 mmol), pyridine (3 mL) and excess LiCl was stirred for 2 h at 80 °C under a positive pressure of argon. The solution was poured into ethanol (150 mL) to precipitate of a white solid. The residue was chromatographed on silica gel with $CHCl_3$/EtOH (99/1 v/v) as an eluent, where the second fraction was collected and evaporated to dryness. The residue was subjected to recycling preparative HPLC (YMC LC-forte/R) with $CHCl_3$ as an eluent at a flow rate of 10 mL min^{-1},

where the first fraction was collected and evaporated to dryness under reduced pressure to give **C_3(*S*)** as white solid (212 mg, 0.11 mmol, 50%). ^{1}H NMR (500 MHz, $CDCl_3$, 25 °C, TMS) δ (ppm) = 7.82 (s, 3H), 7.50 (d, *J* = 9.2 Hz, 6H), 7.07 (m, 12H), 4.05 (m, 18H), 1.92-1.46 (m, 36H), 1.41-1.08 (overlapped m, 54H), 0.93 (m, 27H), 0.87 (d, *J* = 6.3 Hz, 54H); ^{13}C NMR (125 MHz, $CDCl_3$, 25 °C, TMS) δ (ppm) = 165.6, 153.2, 144.2, 141.4, 132.9, 129.8, 124.4, 121.6, 105.7, 71.8, 67.7, 39.3, 37.4, 36.3, 29.8, 29.6, 28.0, 24.7, 22.6, 19.6. MALDI-TOF mass: m/z calculated, for compound **C_3(*S*)** $[M + H]^+$, 2007.59; Found: $[M + H]^+$, 2007.91.

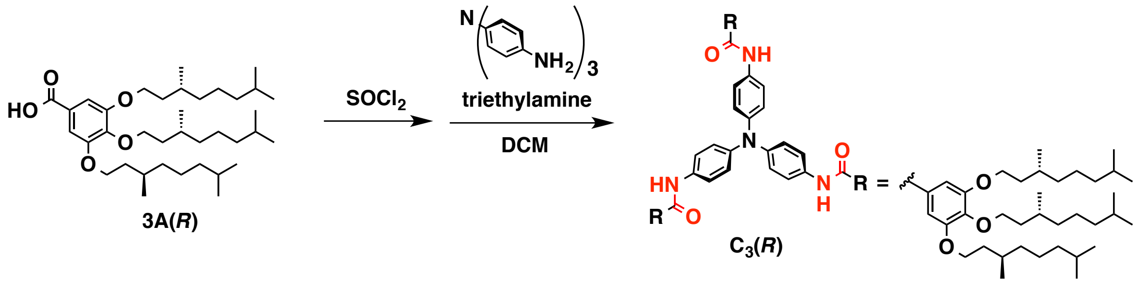

C_3(*R*): A thionyl chloride (10 mL) solution of **3A(*R*)** (207 mg, 0.34 mmol) was refluxed for 6 h, and the reaction mixture was evaporated to dryness. To a NMP solution (20 mL) of the residue were successively added a mixture of tris(4-aminophenyl) amine (28 mg, 0.10 mmol), pyridine (2 mL) and excess LiCl was stirred for 2 h at 80 °C under a positive pressure of argon. The solution was poured into ethanol (100 mL) to precipitate of a white solid. The residue was chromatographed on silica gel with $CHCl_3$/EtOH (99/1 v/v) as an eluent, where the second fraction was collected and evaporated to dryness. The residue was subjected to recycling preparative HPLC (YMC LC-forte/R) with $CHCl_3$ as an eluent at a flow rate of 10 mL min^{-1}, where the first fraction was collected and evaporated to dryness under reduced pressure to give **C_3(*R*)** as white solid (109 mg, 0.054 mmol, 54%). ^{1}H NMR (500 MHz, $CDCl_3$, 25 °C, TMS) δ (ppm) = 7.68 (s, 3H), 7.51 (d, *J* = 9.2 Hz, 6H), 7.08 (m, 12H), 4.05 (m, 18H), 1.87-1.47 (m, 36H), 1.42-1.09 (overlapped m, 54H), 0.94 (m, 27H), 0.86 (d, *J* = 6.3 Hz, 54H); ^{13}C NMR (125 MHz, $CDCl_3$, 25 °C, TMS) δ (ppm) = 165.5, 153.3, 144.3, 141.5, 132.9, 129.8, 124.4, 121.5, 105.7, 71.8, 67.8, 39.3, 37.4, 36.4, 29.8, 29.6, 28.0, 24.7, 22.6, 19.6. MALDI-TOF mass: m/z calculated, for compound **C_3(*R*)** $[M + H]^+$, 2007.59; Found: $[M + H]^+$, 2007.65.

3. Analytical Data

3.1. ^{1}H and ^{13}C NMR Spectroscopy

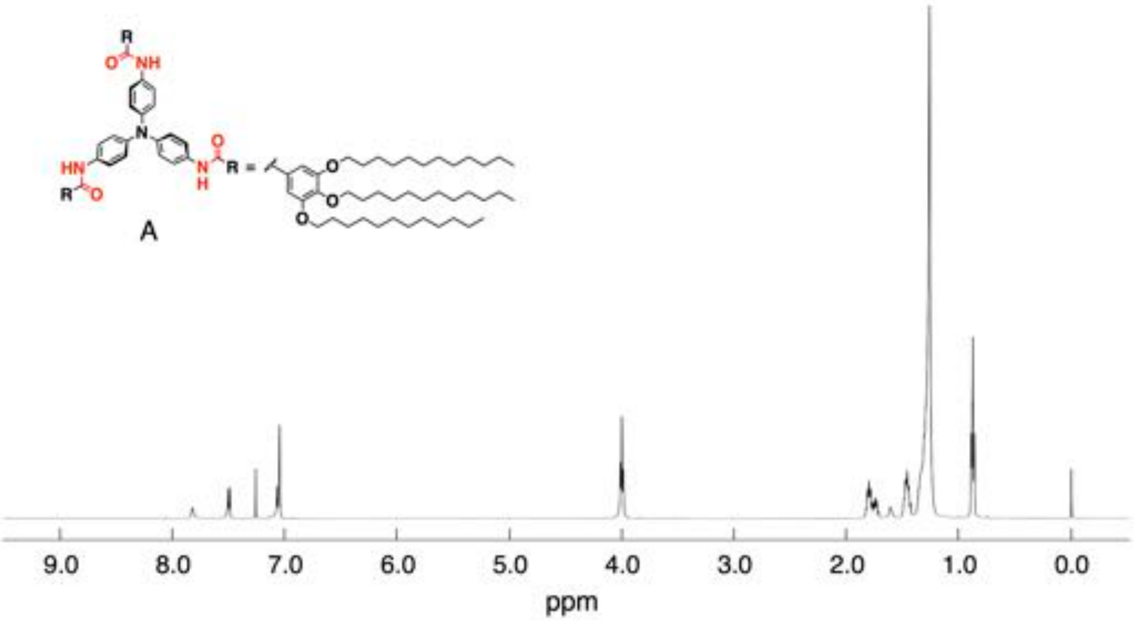

Figure S1. (a) ^{1}H NMR spectrum (500 MHz) of **A** in $CDCl_3$ at 25 °C.

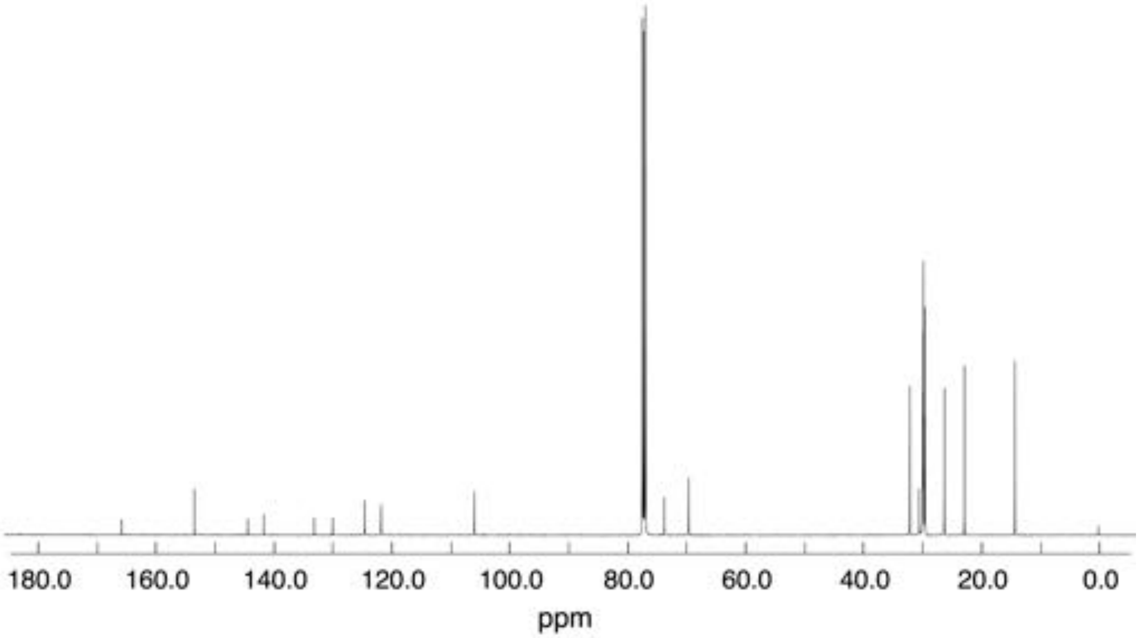

Figure S1. (b) ^{13}C NMR spectrum (125 MHz) of **A** in $CDCl_3$ at 25 °C.

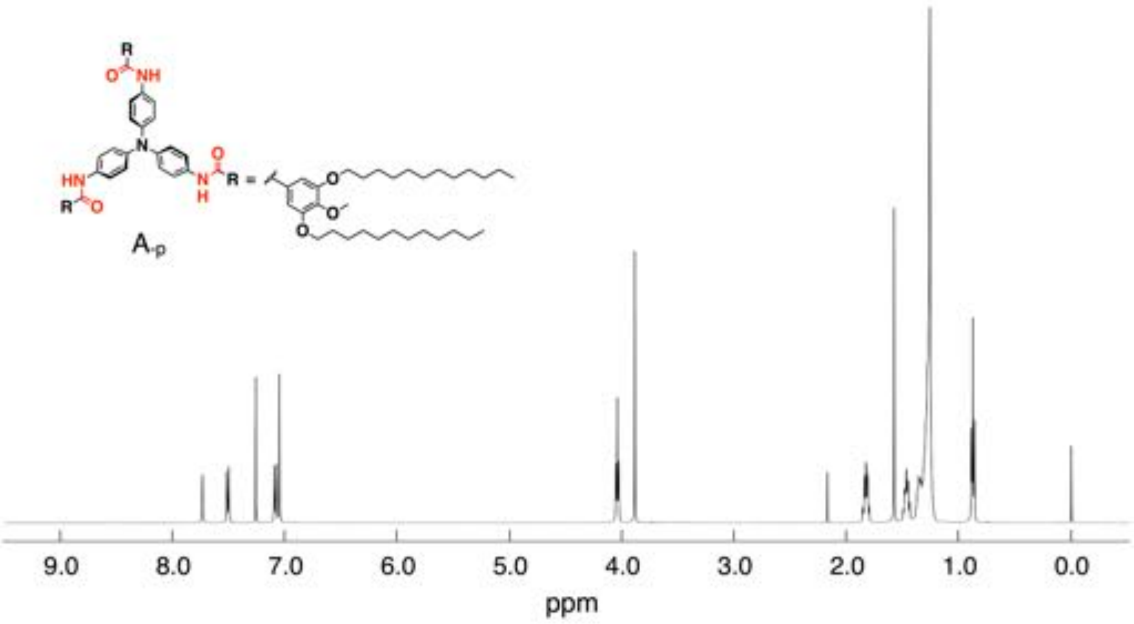

Figure S2. (a) ^{1}H NMR spectrum (500 MHz) of **A-p** in $CDCl_3$ at 25 °C.

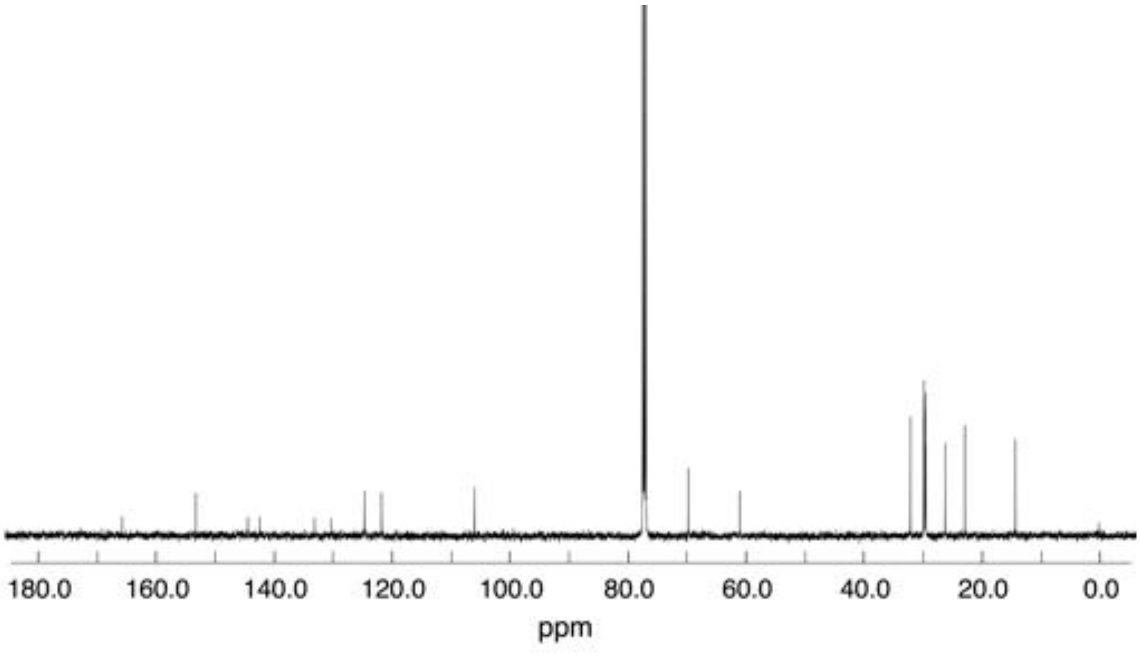

Figure S2. (b) ^{13}C NMR spectrum (125 MHz) of **A-p** in $CDCl_3$ at 25 °C.

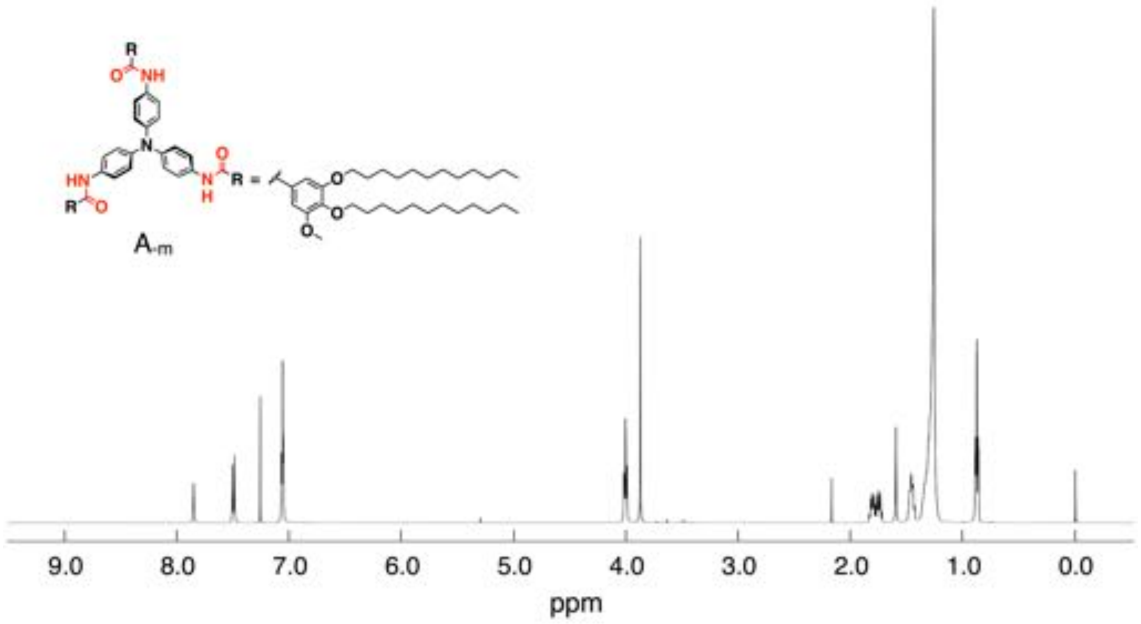

Figure S3. (a) ^{1}H NMR spectrum (500 MHz) of **A-m** in $CDCl_3$ at 25 °C.

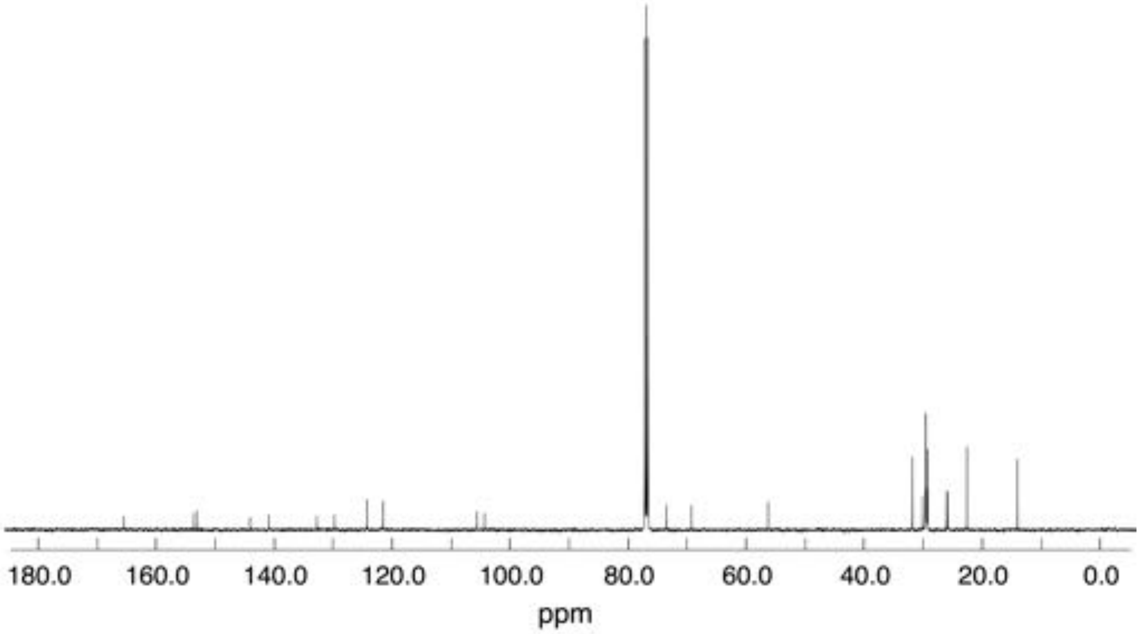

Figure S3. (b) ^{13}C NMR spectrum (125 MHz) of **A-m** in $CDCl_3$ at 25 °C.

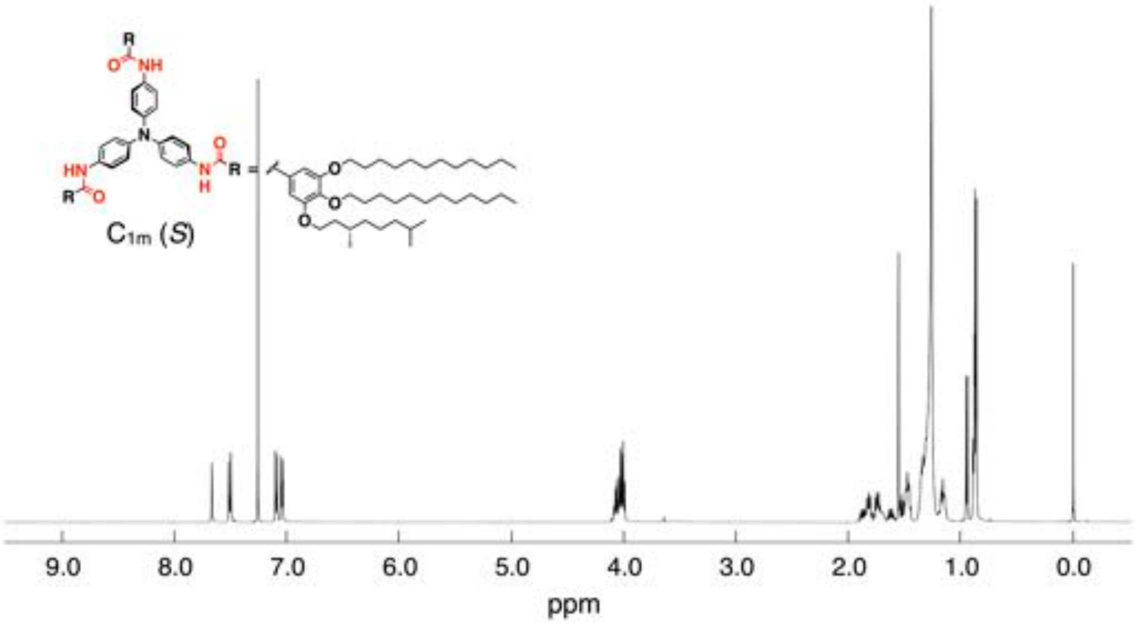

Figure S4. (a) ^{1}H NMR spectrum (500 MHz) of $\mathbf{C_{1m}}$(***S***) in $CDCl_3$ at 25 °C.

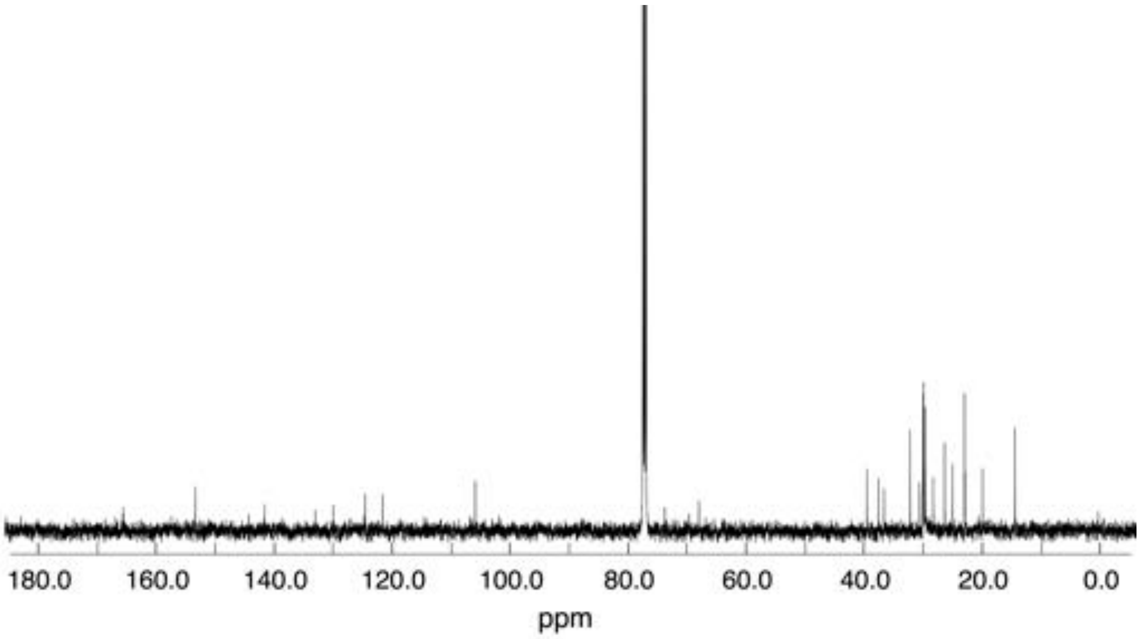

Figure S4. (b) ^{13}C NMR spectrum (125 MHz) of $\mathbf{C_{1m}}$(***S***) in $CDCl_3$ at 25 °C.

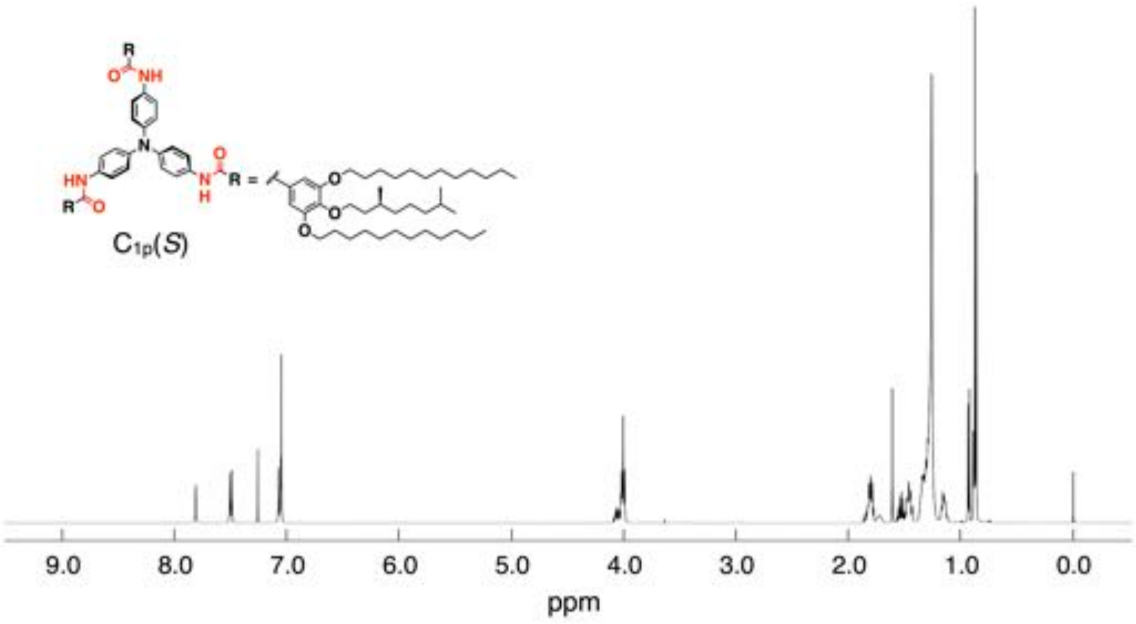

Figure S5. (a) ^{1}H NMR spectrum (500 MHz) of **C_{1p}(*S*)** in $CDCl_3$ at 25 °C.

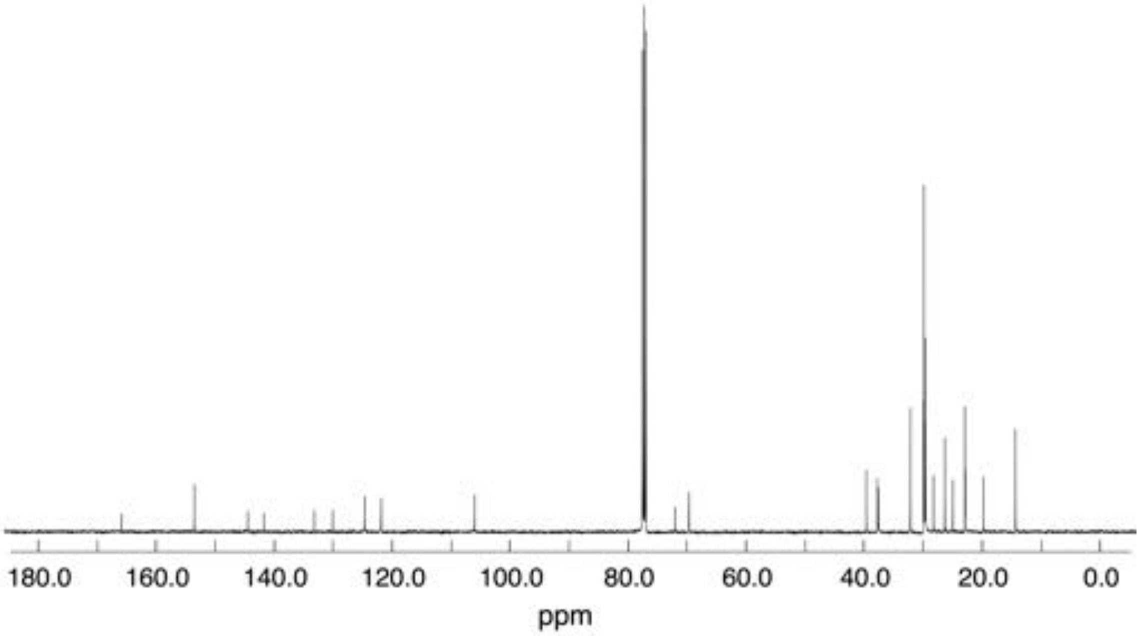

Figure S5. (b) ^{13}C NMR spectrum (125 MHz) of **C_{1p}(*S*)** in $CDCl_3$ at 25 °C.

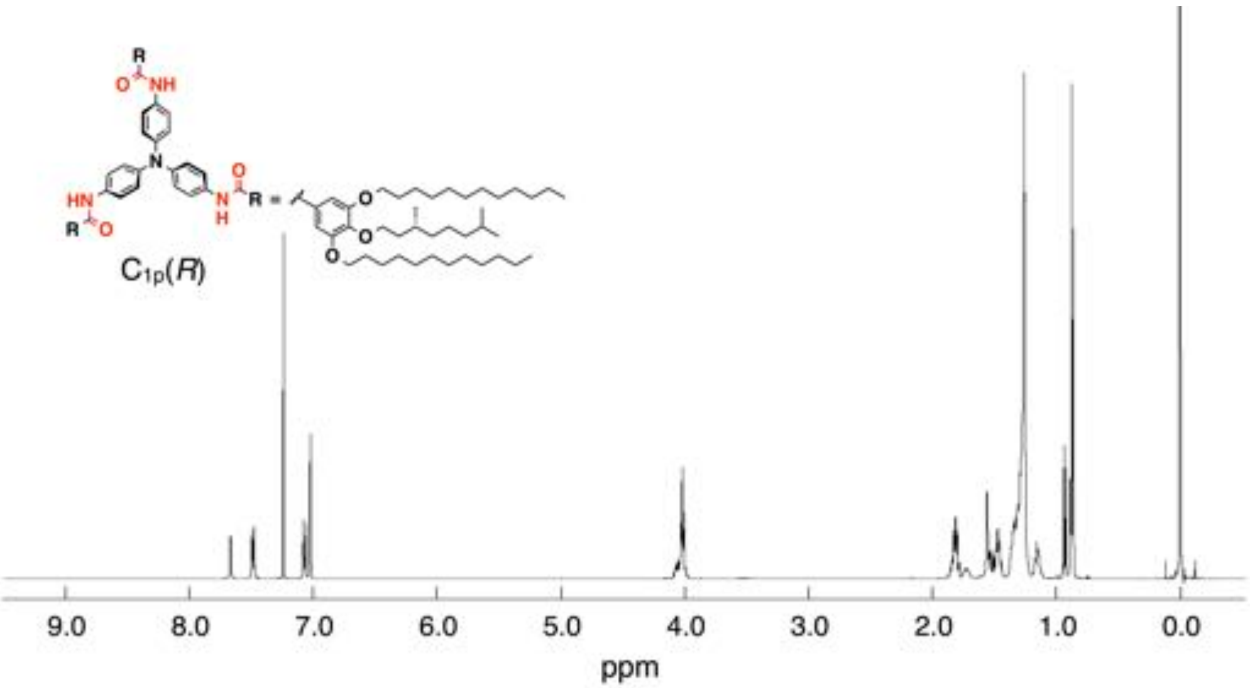

Figure S6. (a) ^{1}H NMR spectrum (500 MHz) of **C_{1p}(*R*)** in $CDCl_3$ at 25 °C.

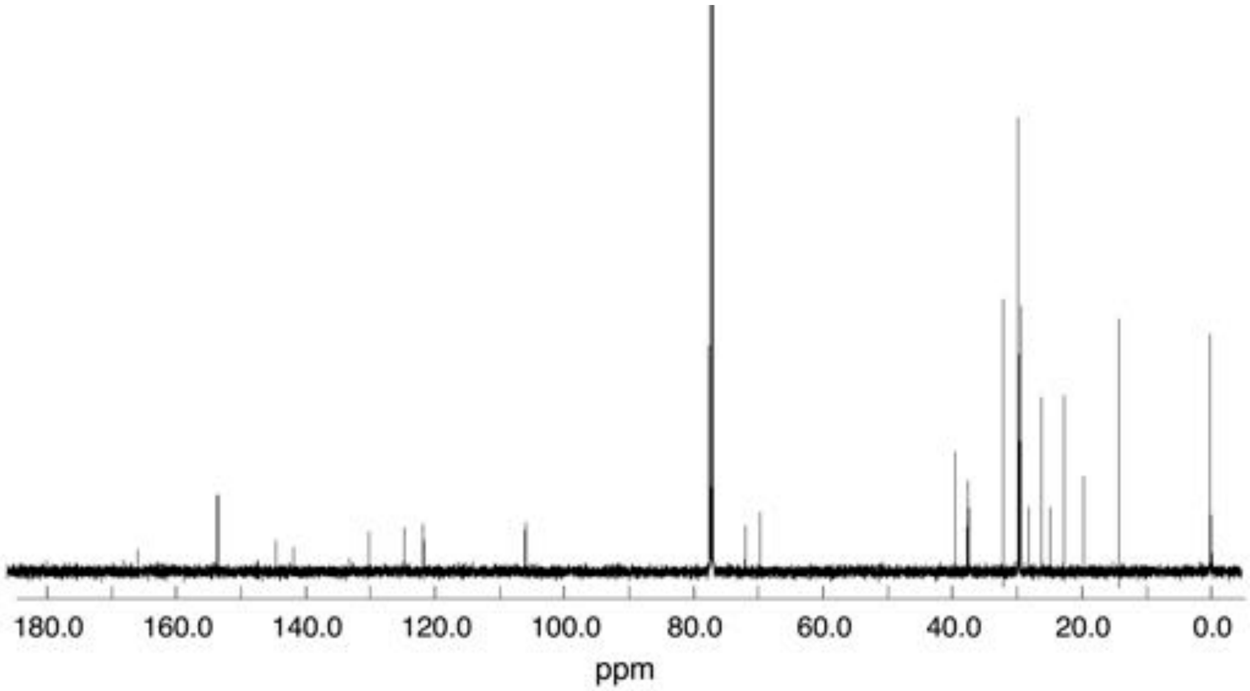

Figure S6. (b) ^{13}C NMR spectrum (125 MHz) of **C_{1p}(*R*)** in $CDCl_3$ at 25 °C.

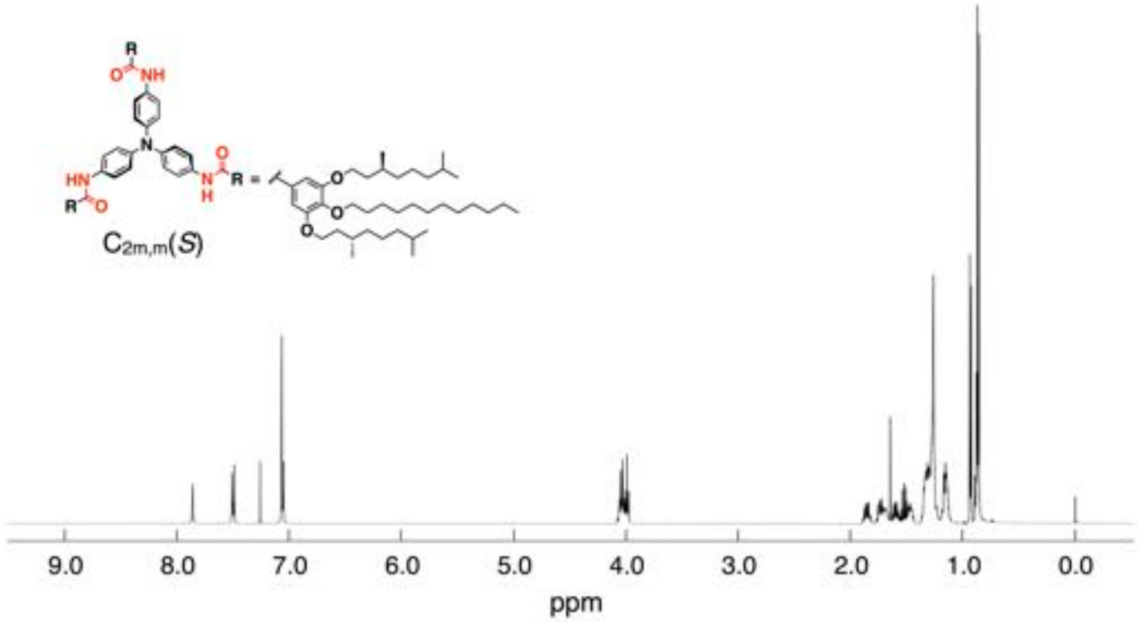

Figure S7. (a) ^{1}H NMR spectrum (500 MHz) of **$C_{2m,m}$(*S*)** in $CDCl_3$ at 25 °C.

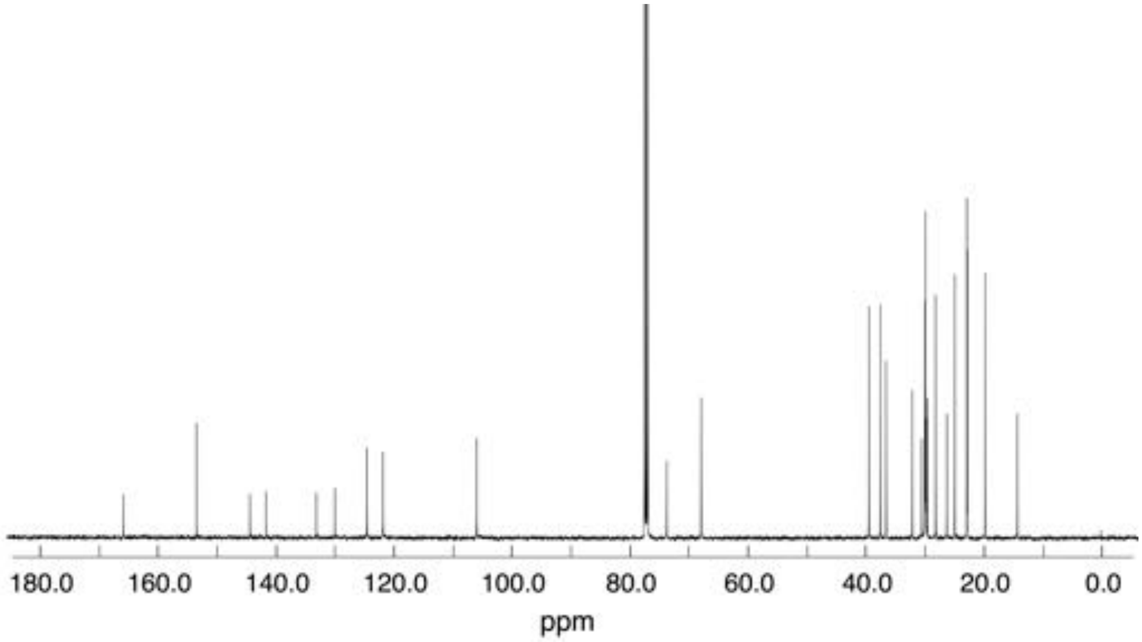

Figure S7. (b) ^{13}C NMR spectrum (125 MHz) of **$C_{2m,m}$(*S*)** in $CDCl_3$ at 25 °C.

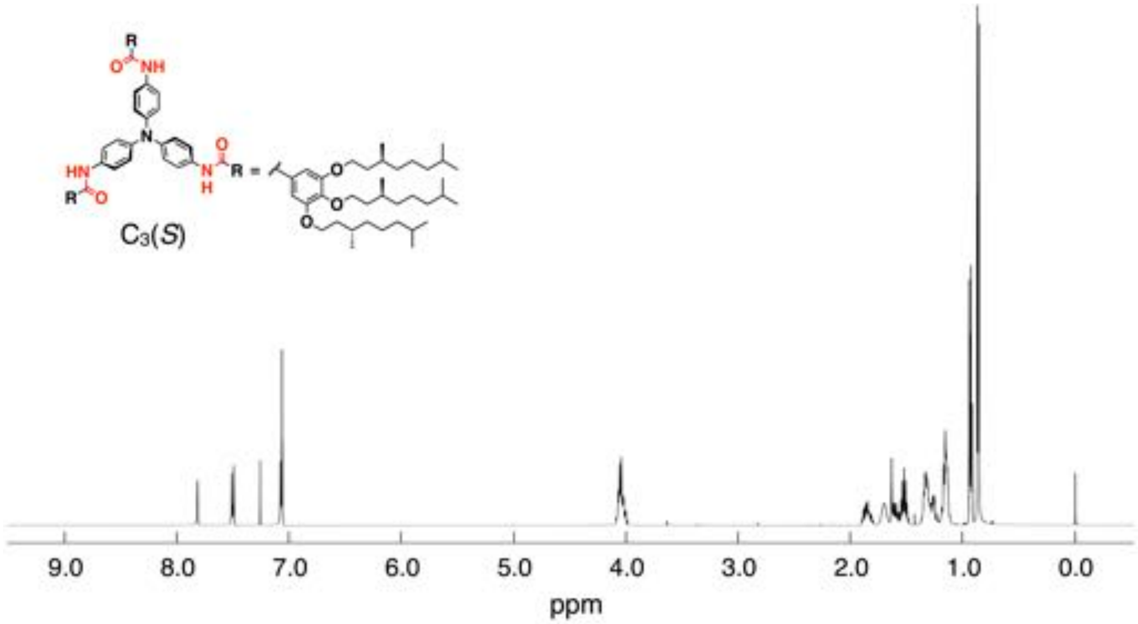

Figure S8. (a) ^{1}H NMR spectrum (500 MHz) of **C_3(*S*)** in $CDCl_3$ at 25 °C.

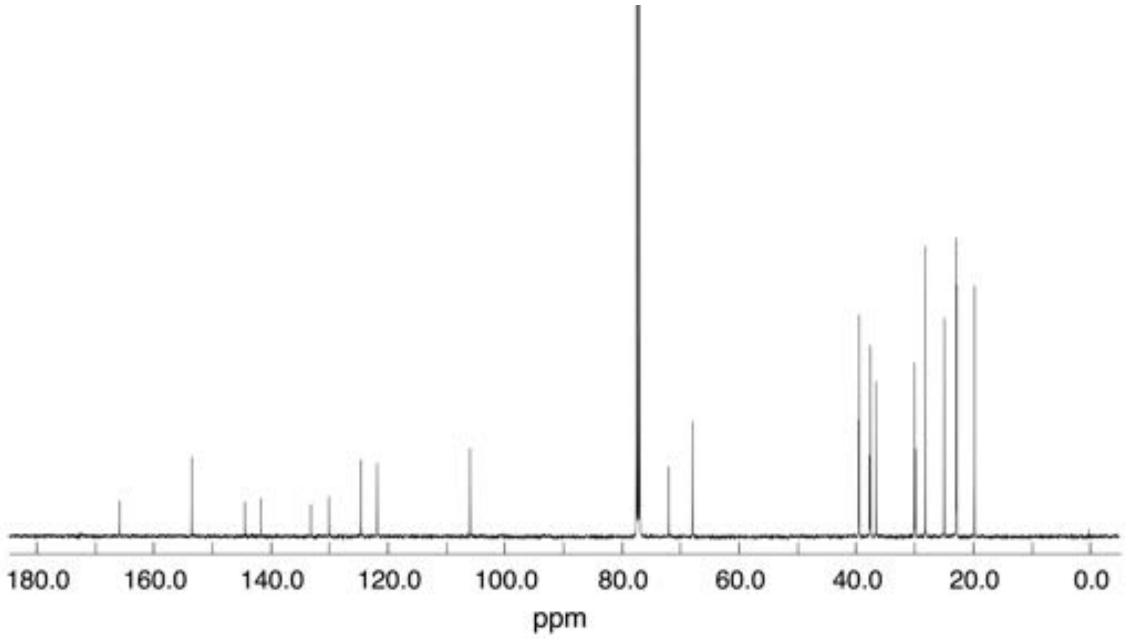

Figure S8. (b) ^{13}C NMR spectrum (125 MHz) of **C_3(*S*)** in $CDCl_3$ at 25 °C.

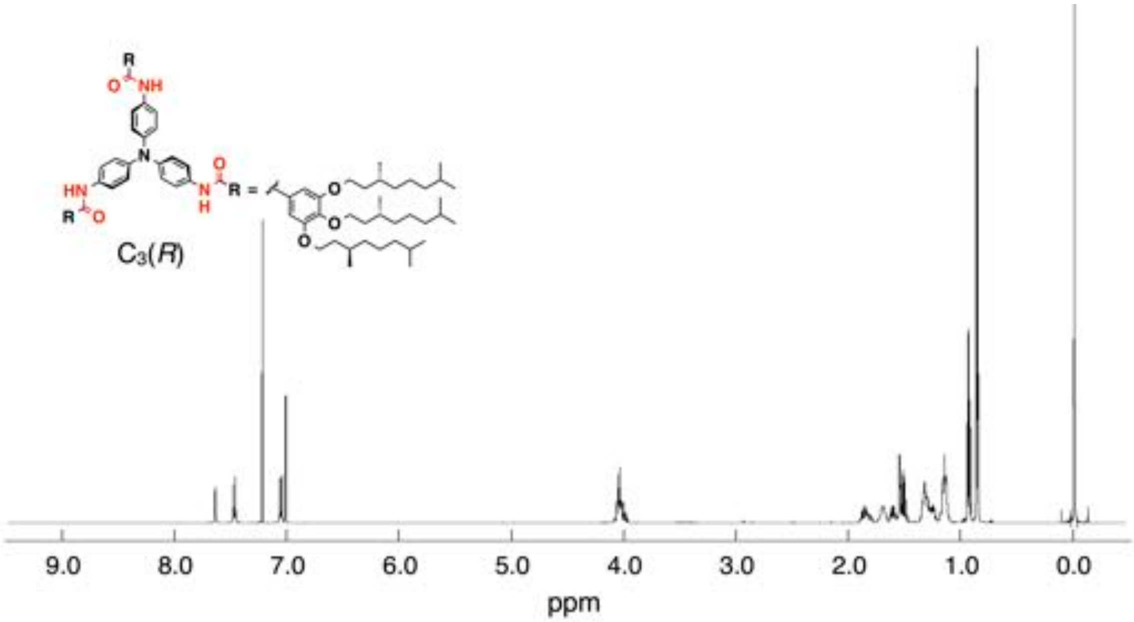

Figure S9. (a) ^{1}H NMR spectrum (500 MHz) of **C_3(*R*)** in $CDCl_3$ at 25 °C.

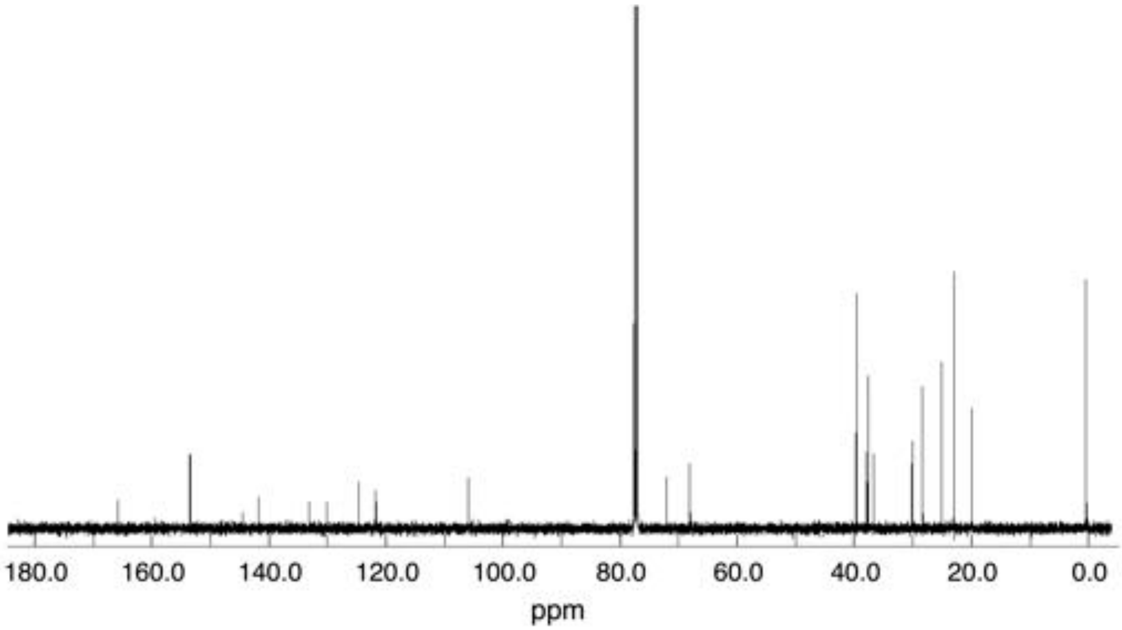

Figure S9. (b) ^{13}C NMR spectrum (125 MHz) of **C_3(*R*)** in $CDCl_3$ at 25 °C.

3.2. MALDI-TOF Mass spectrometry

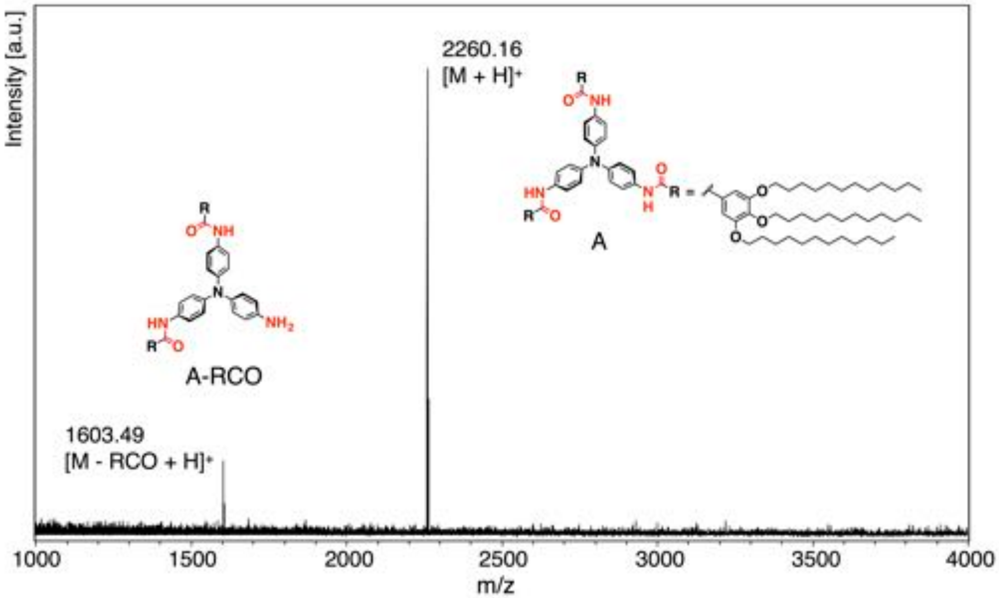

Figure S10. MALDI-TOF-MS spectrum of **A** using CHCA as a matrix. The peak due to [M – RCO + H]$^+$ became more intense when a larger laser power was applied. Therefore, the laser power was adjusted to be as small as possible in order to avoid fragmentation.

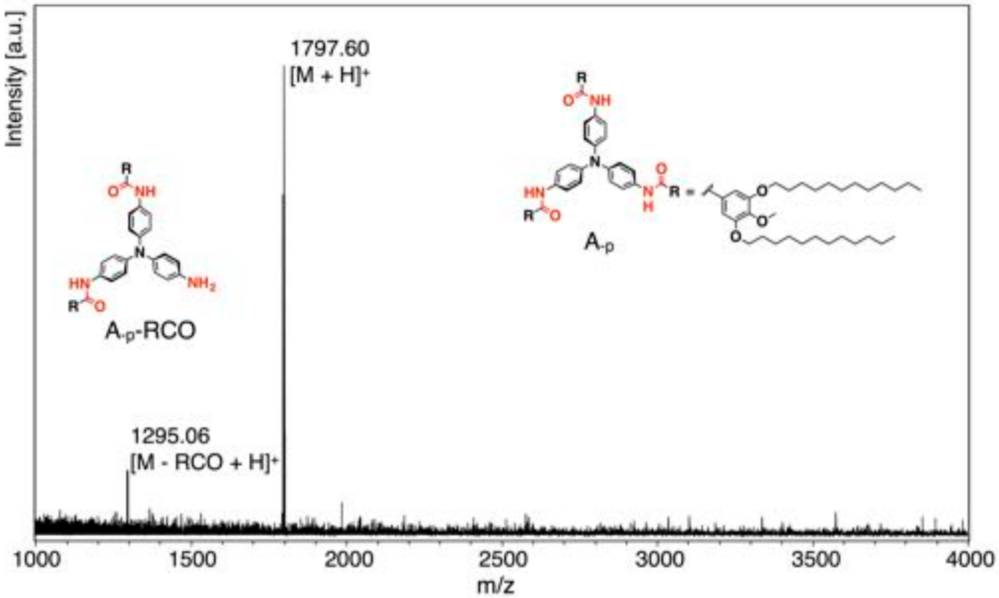

Figure S11. MALDI-TOF-MS spectrum of $\mathbf{A}_{\text{-p}}$ using CHCA as a matrix. The peak due to [M – RCO + H]$^+$ became more intense when a larger laser power was applied. Therefore, the laser power was adjusted to be as small as possible in order to avoid fragmentation.

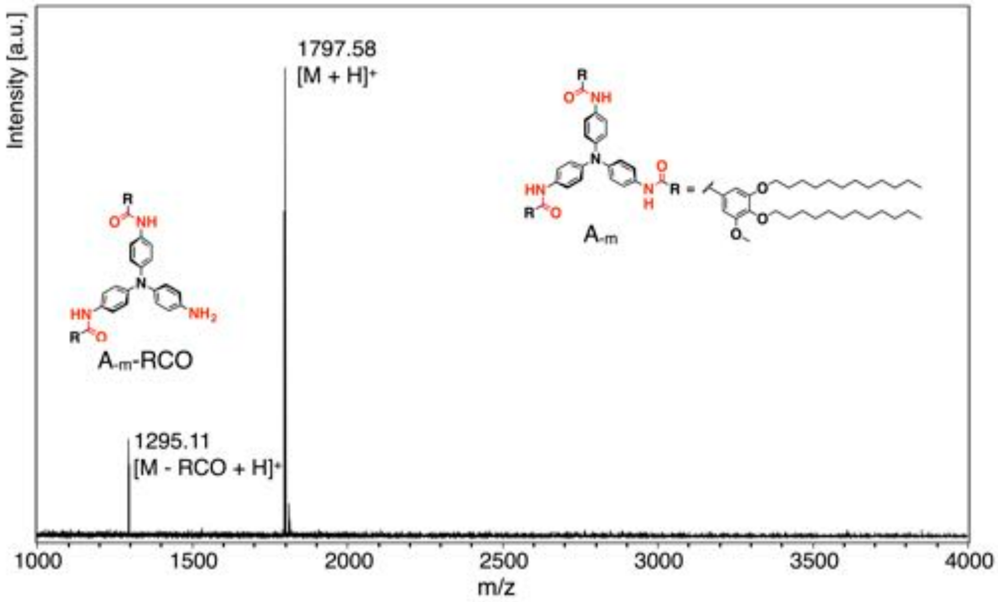

Figure S12. MALDI-TOF-MS spectrum of $\mathbf{A_{-m}}$ using CHCA as a matrix. The peak due to [M – RCO + H]$^+$ became more intense when a larger laser power was applied. Therefore, the laser power was adjusted to be as small as possible in order to avoid fragmentation.

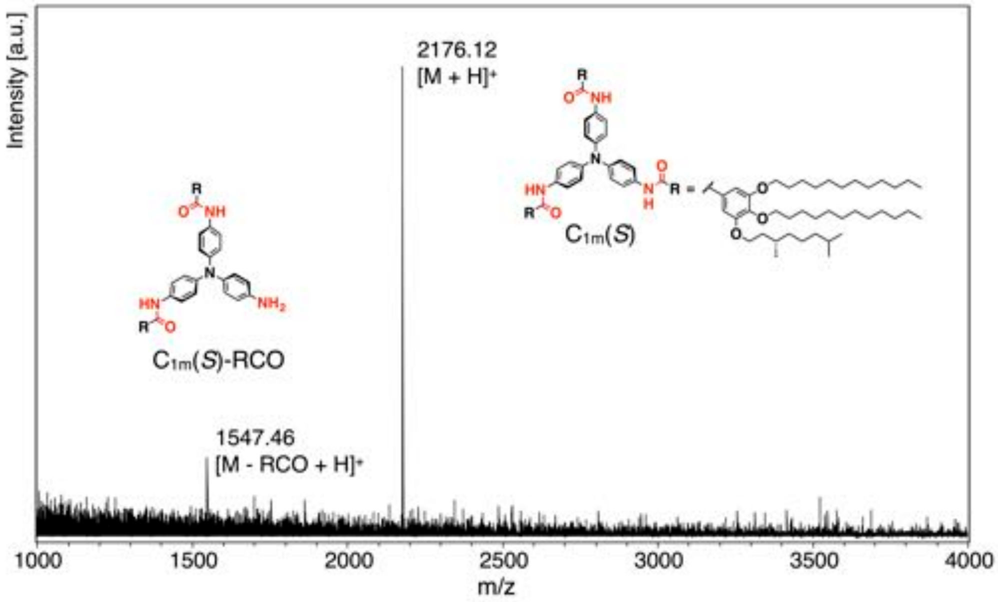

Figure S13. MALDI-TOF-MS spectrum of $\mathbf{C_{1m}}$(***S***) using CHCA as a matrix. The peak due to [M – RCO + H]$^+$ became more intense when a larger laser power was applied. Therefore, the laser power was adjusted to be as small as possible in order to avoid fragmentation.

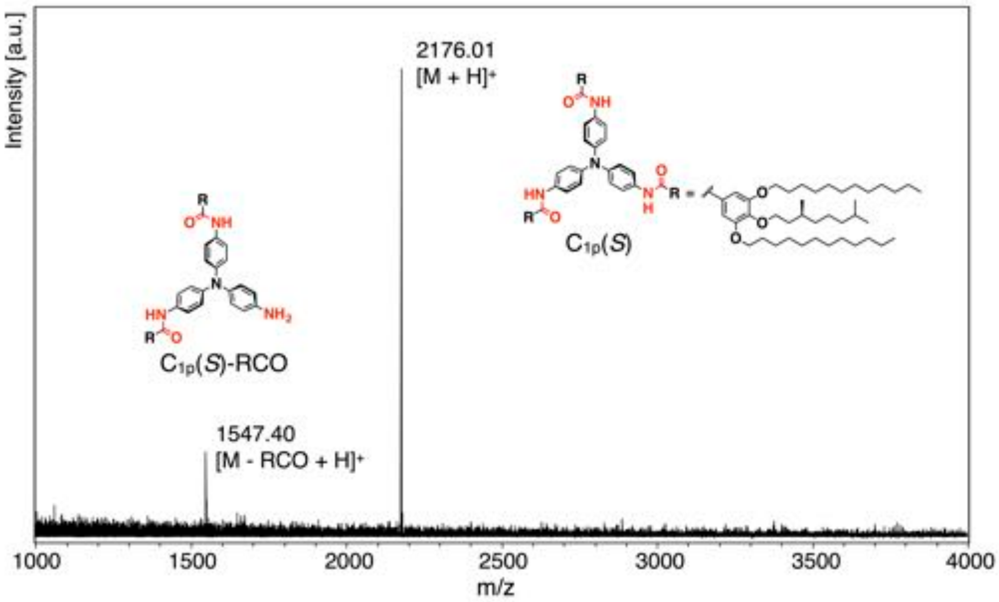

Figure S14. MALDI-TOF-MS spectrum of $\mathbf{C_{1p}}$(***S***) using CHCA as a matrix. The peak due to [M – RCO + H]$^+$ became more intense when a larger laser power was applied. Therefore, the laser power was adjusted to be as small as possible in order to avoid fragmentation.

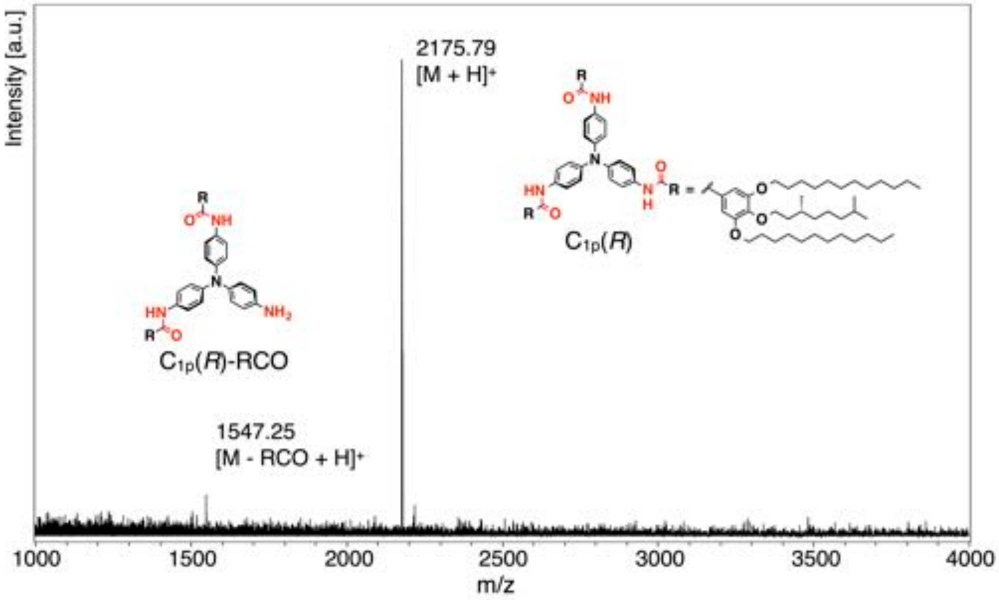

Figure S15. MALDI-TOF-MS spectrum of $\mathbf{C_{1p}}$(***R***) using CHCA as a matrix. The peak due to [M – RCO + H]$^+$ became more intense when a larger laser power was applied. Therefore, the laser power was adjusted to be as small as possible in order to avoid fragmentation.

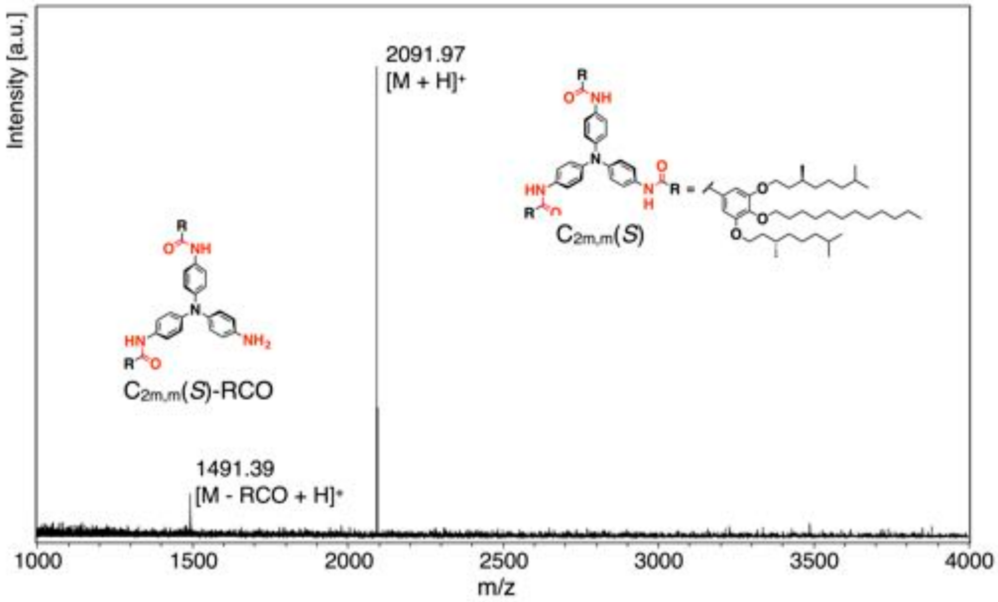

Figure S16. MALDI-TOF-MS spectrum of $\mathbf{C_{2m,m}}$(*S*) using CHCA as a matrix. The peak due to [M – RCO + H]$^+$ became more intense when a larger laser power was applied. Therefore, the laser power was adjusted to be as small as possible in order to avoid fragmentation.

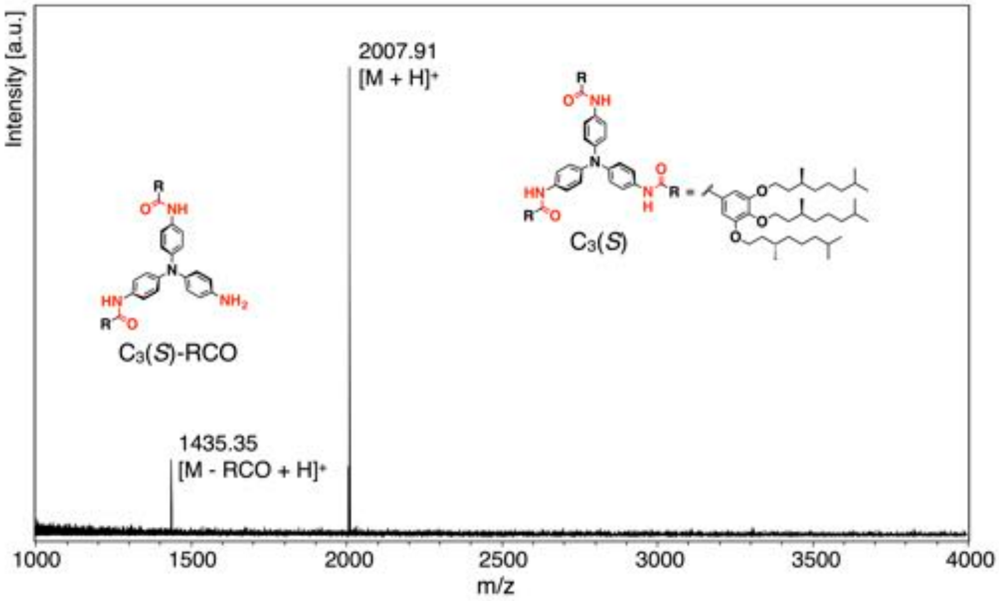

Figure S17. MALDI-TOF-MS spectrum of $\mathbf{C_3}$(*S*) using CHCA as a matrix. The peak due to [M – RCO + H]$^+$ became more intense when a larger laser power was applied. Therefore, the laser power was adjusted to be as small as possible in order to avoid fragmentation.

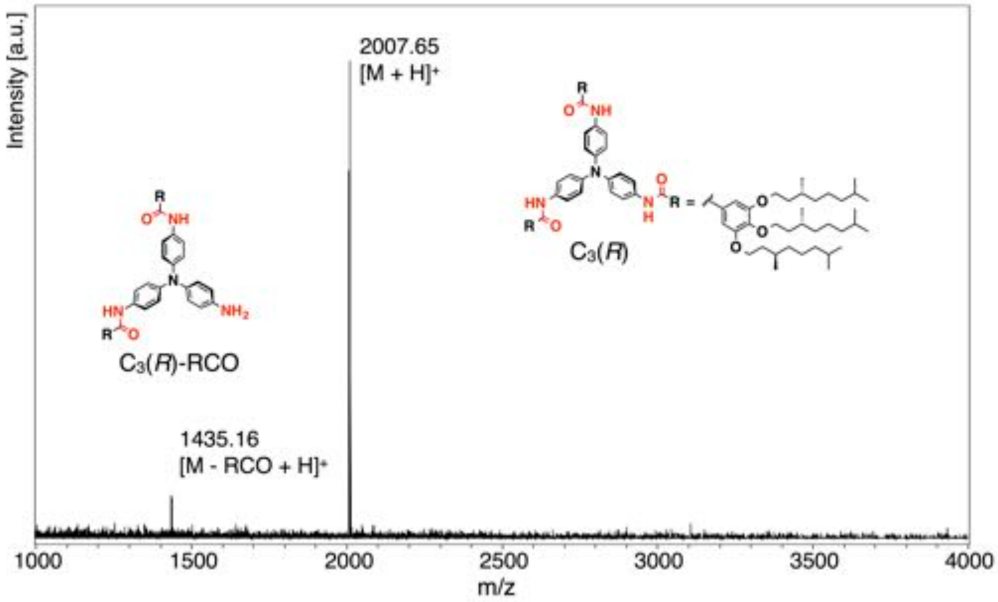

Figure S18. MALDI-TOF-MS spectrum of $\mathbf{C_3(\mathit{R})}$ using CHCA as a matrix. The peak due to [M – RCO + H]$^+$ became more intense when a larger laser power was applied. Therefore, the laser power was adjusted to be as small as possible in order to avoid fragmentation.

4. Supplementary Figures

4.1. DLS and Electronic Absorption Profiles of A

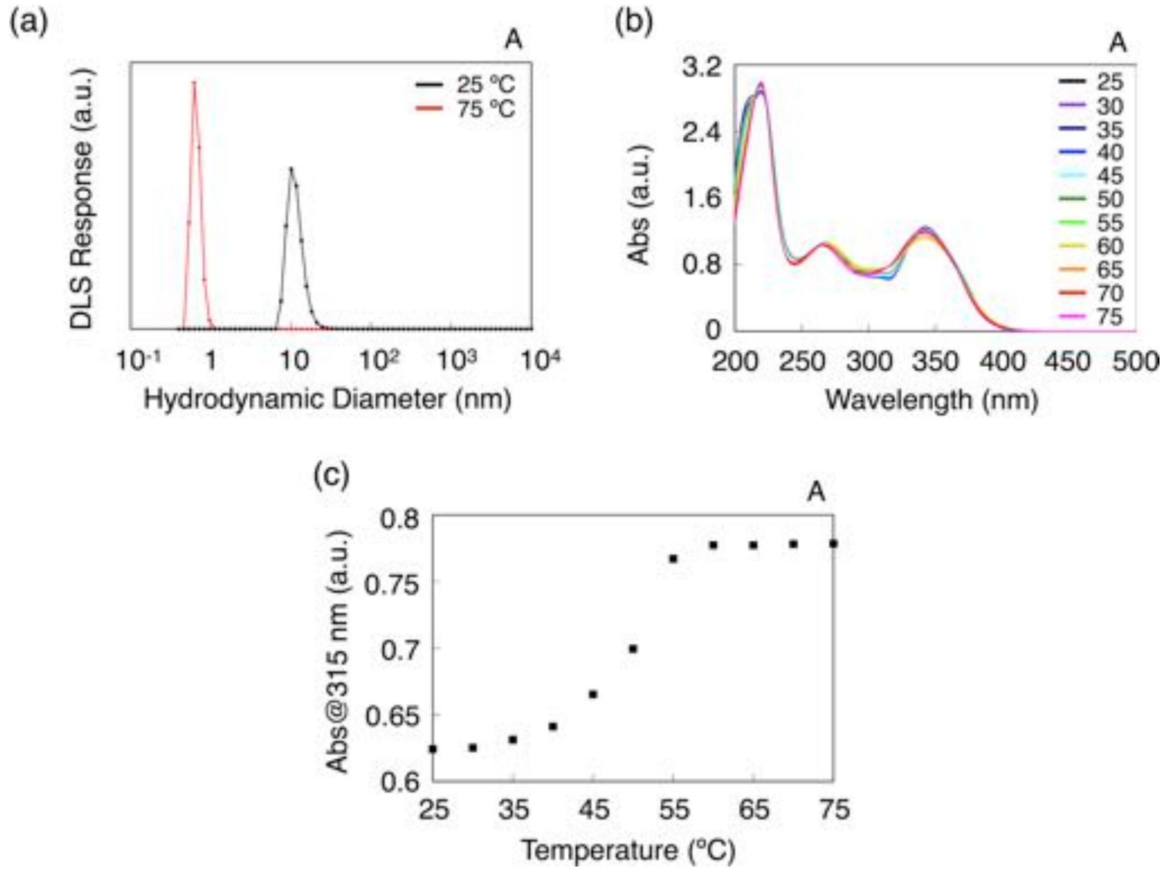

Figure S19. (a) Dynamic light scattering (DLS) profiles and (b) electronic absorption spectra of **A** (30 μM) in cyclohexane at 25 °C (black) and 75 °C (red). (c) Plots of the absorbance at 315 nm in the electronic absorption spectra of **A** (30 μM) in cyclohexane during the cooling process from 75 °C to 25 °C.

4.2. AFM Height Image Analysis of Polymerized A

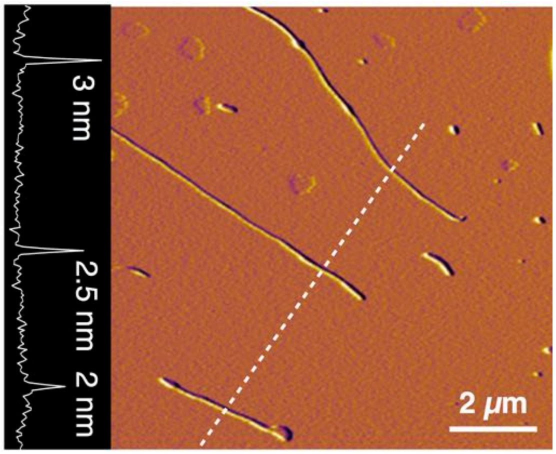

Figure S20. Tapping-mode AFM image on a silicon substrate with the height profile of air-dried 30 μM cyclohexane solution of **A** at 25 °C.

4.3. Electronic Absorption Spectra of TPA Derivatives

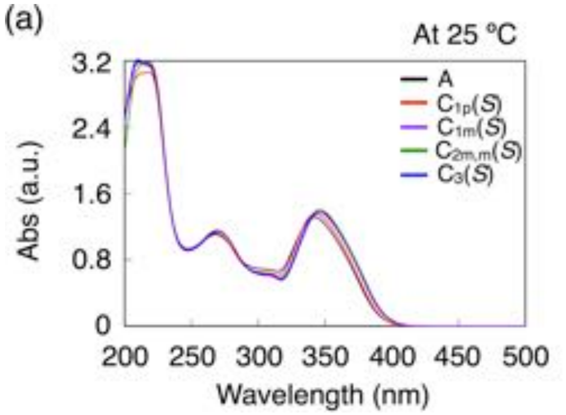

Figure S21. Electronic absorption spectra of (a) chiral TPAs: $\mathbf{C_{1p}}$(***S***), $\mathbf{C_{1m}}$(***S***), $\mathbf{C_{2m,m}}$(***S***), $\mathbf{C_{3}}$(***S***), and (b) achiral TPAs: **A**, $\mathbf{A_{-p}}$, $\mathbf{A_{-m}}$ at 25 ºC in cyclohexane. All sample concentrations were 30 μM.

4.4. FT-IR spectra of C_3(***R***), C_3(***S***) and 9:1 Mixtures of A/C_3(***R***) and A/C_3(***S***)

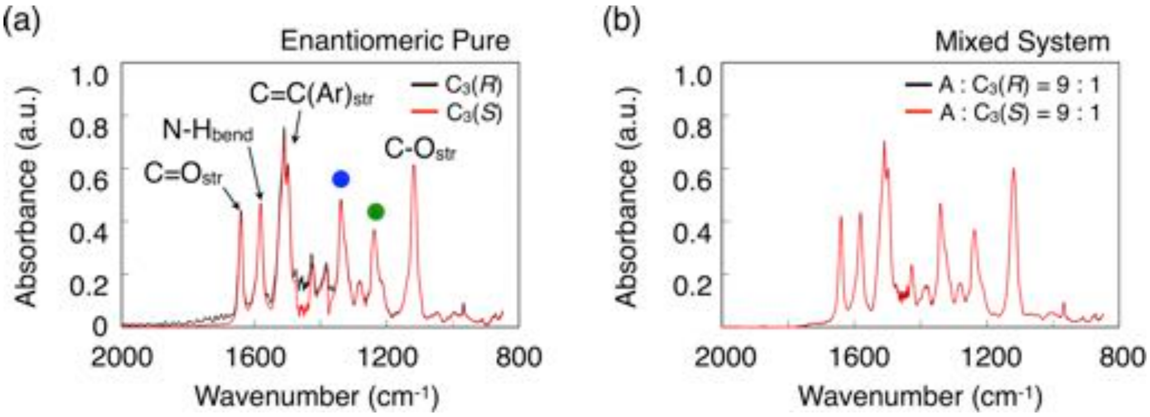

Figure S22. FT-IR spectra of (a) **C_3(*R*)**, **C_3(*S*)** and (b) 9:1 mixtures of **A/C_3(*R*)** and **A/C_3(*S*)** in methylcyclohexane at 25 °C. The stretching vibration bands at 1322 cm^{-1} (blue) and 1242 cm^{-1} (green) were assigned as the C-N vibrational modes of the outer amine linked with TPA and the central amine present in TPA, respectively. Individual absorption bands were assigned as described in a previous report (see the manuscript). All sample concentrations were 5 mM.

4.5. Sergeants and Soldiers Experiments for Mixtures of $A/C_3(S)$ (1)

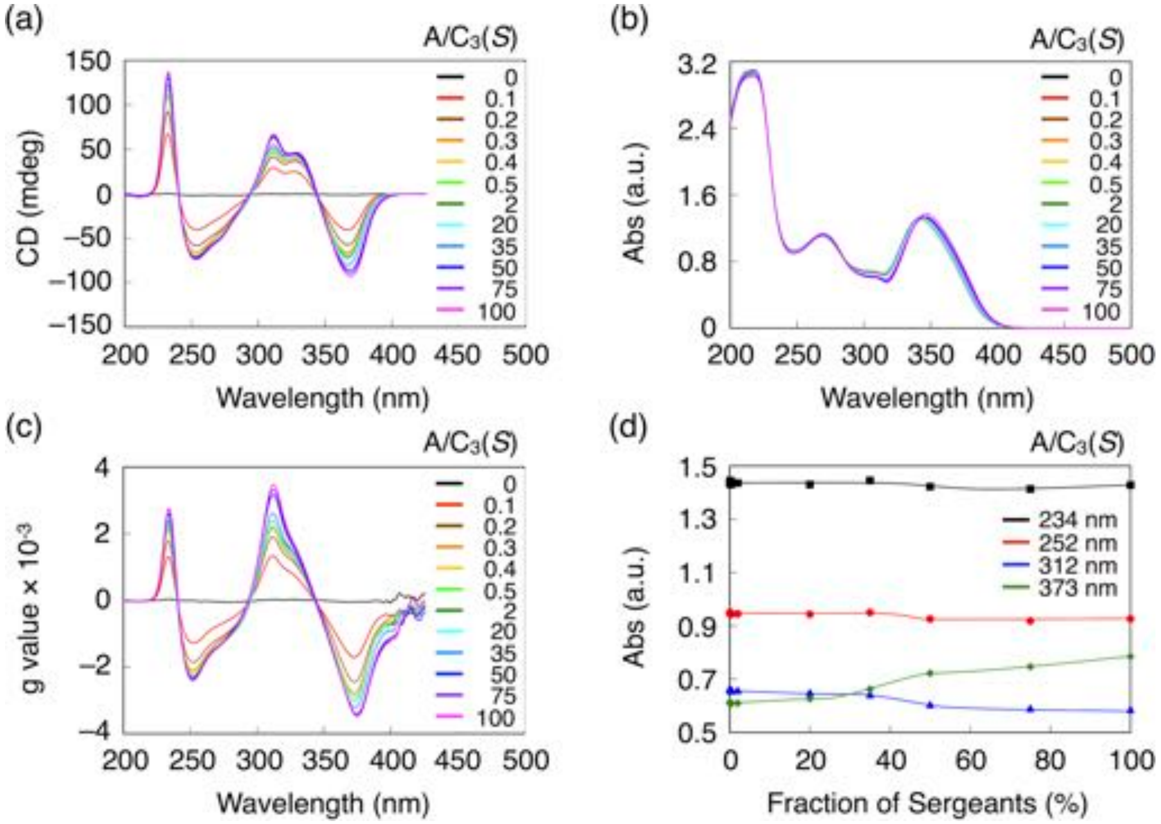

Figure S23. (a) CD, (b) electronic absorption, and (c) g-value spectra for the mixtures of $\mathbf{A/C_3}$(***S***) containing various mole fractions of $\mathbf{C_3}$(***S***) at a total concentration of 30 μM at 25 ºC in cyclohexane. (d) Plots of the absorbance at 234, 252, 312, and 373 nm obtained by (b) against the fraction of sergeants.

4.6. Sergeants and Soldiers Experiments for Mixtures of A/C_3(*S*) (2)

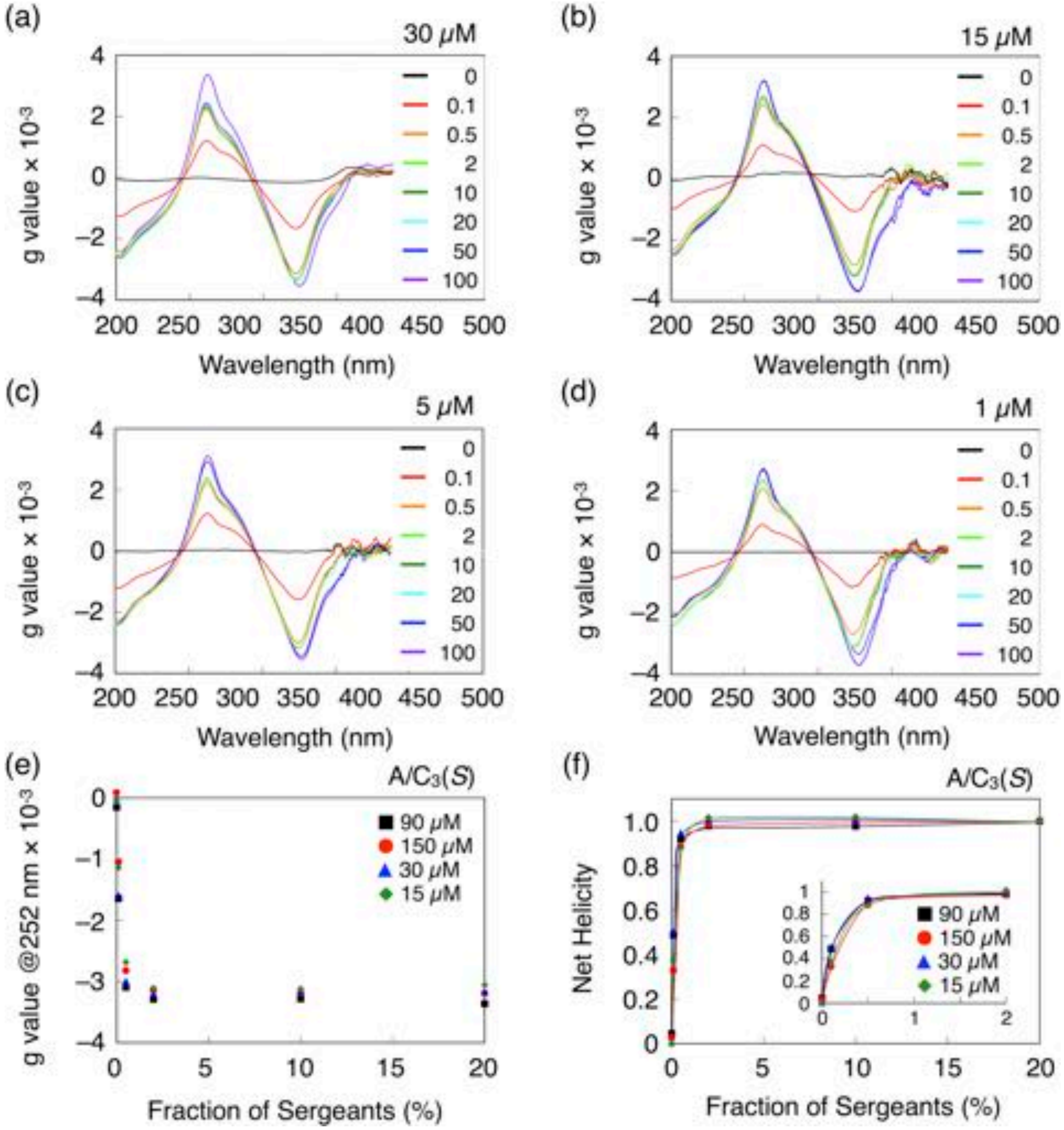

Figure S24. g-value spectra for the mixtures of **A/C_3(*S*)** containing various mole fractions of **C_3(*S*)** at total concentrations of (a) 30 μM, (b) 15 μM, (c) 5 μM, and (d) 1 μM at 25 ºC in cyclohexane. (f) Plots of net helicity against the fraction of the sergeants for the mixtures of **A/C_3(*S*)** at total concentrations of 30 (red), 15 (black), 5 (blue), and 1 μM (green).

4.7. Theoretical Estimation of the MMP and HRP values

The MMP and HRP values were estimated using the data obtained from the sergeants and soldiers experiments with mixtures of **A/C$_3$(*S*)**. The g-values at 252 nm were converted into the dimensionless net helicity and fit to the least-squares model developed by van der Schoot.[S8] The contour plot of the sum of squared residuals exhibited a narrow region with a minimum at $\sigma = 6.3 \cdot 10^{-8}$ and $\omega = 0.68$ (Figure S19a), and the corresponding fit is shown in Figure S19b. At this point, a combination of HRP and MMP resulted in the best fit of the data.

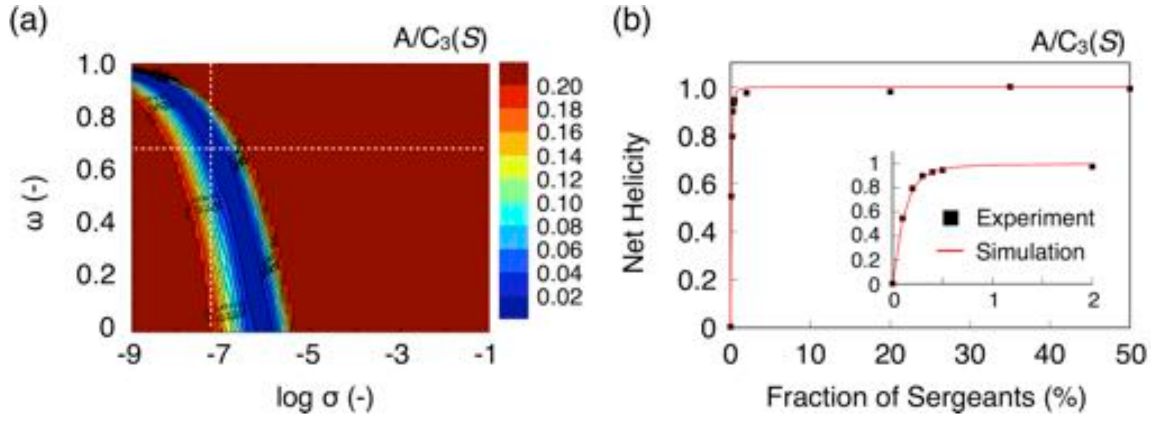

Figure S25. (a) Contour plot of the sum of squared residuals obtained by fitting the g-values for the sergeant and soldiers experiments with mixtures of 30 μM **A/C$_3$(*S*)** in cyclohexane. The minimum point is located where the white dotted lines cross ($\sigma = 6.3 \cdot 10^{-8}$ and $\omega = 0.68$). (b) Comparison of the experimental data and the theoretical fitting curve for the net helicity obtained by the above model.

Table S1. Energy penalties determined from fitting the sergeants and soldiers data.

Molecular core	HRP (kJ mol^{-1})	MMP (kJ mol^{-1})
**Triphenylamine**	20.5	1.0
**Benzene tricarboxamide (BTA)**[S8]	12.6	> 0.5
**Extended BTA**[S5]	7.8[a]	0.94[a]
**Porphyrin**[S9]	16.6	0.2

[a] From fitting the majority-rules experiments

4.8. Sergeants and Soldiers Experiments for Mixtures of A/C_{1p}(*S*)

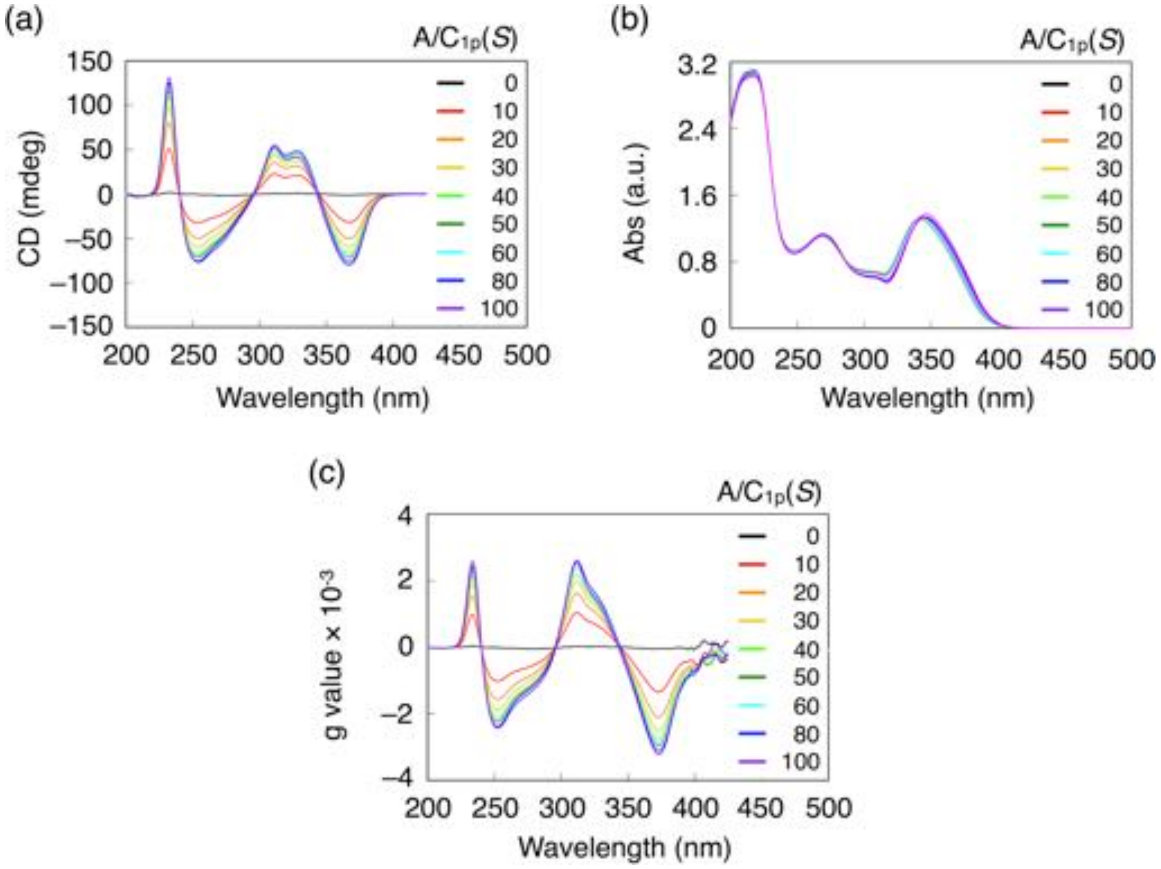

Figure S26. (a) CD, (b) electronic absorption, and (c) g-value spectra for mixtures of **A/C_{1p}(*S*)** containing various mole fractions of the sergeants at a total concentration of 30 μM at 25 °C in cyclohexane.

4.9. Sergeants and Soldiers Experiments for Mixtures of A/C_{1m}(*S*)

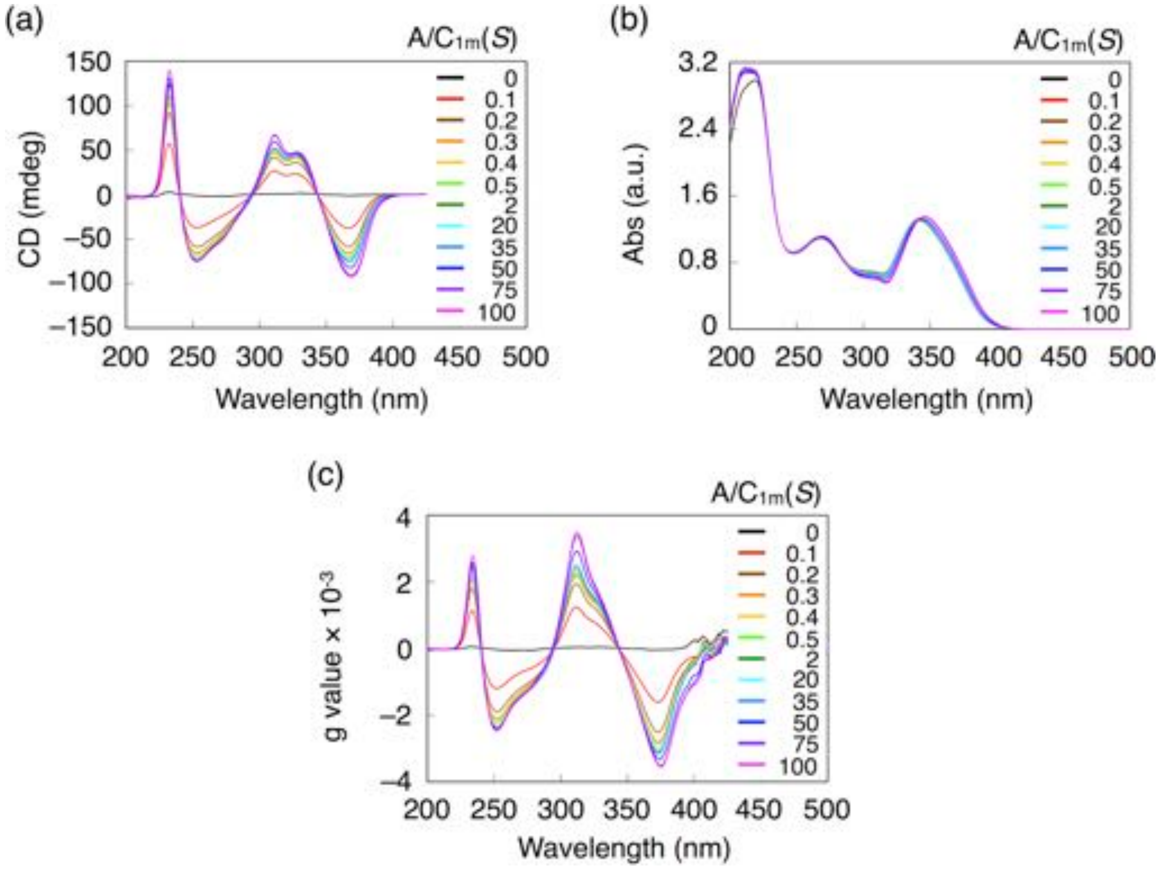

Figure S27. (a) CD, (b) electronic absorption, and (c) g-value spectra for mixtures of **A/C_{1m}(*S*)** containing various mole fractions of the sergeants at a total concentration of 30 μM at 25 °C in cyclohexane.

4.10. Sergeants and Soldiers Experiments for Mixtures of A/C$_{2m,m}$(*S*)

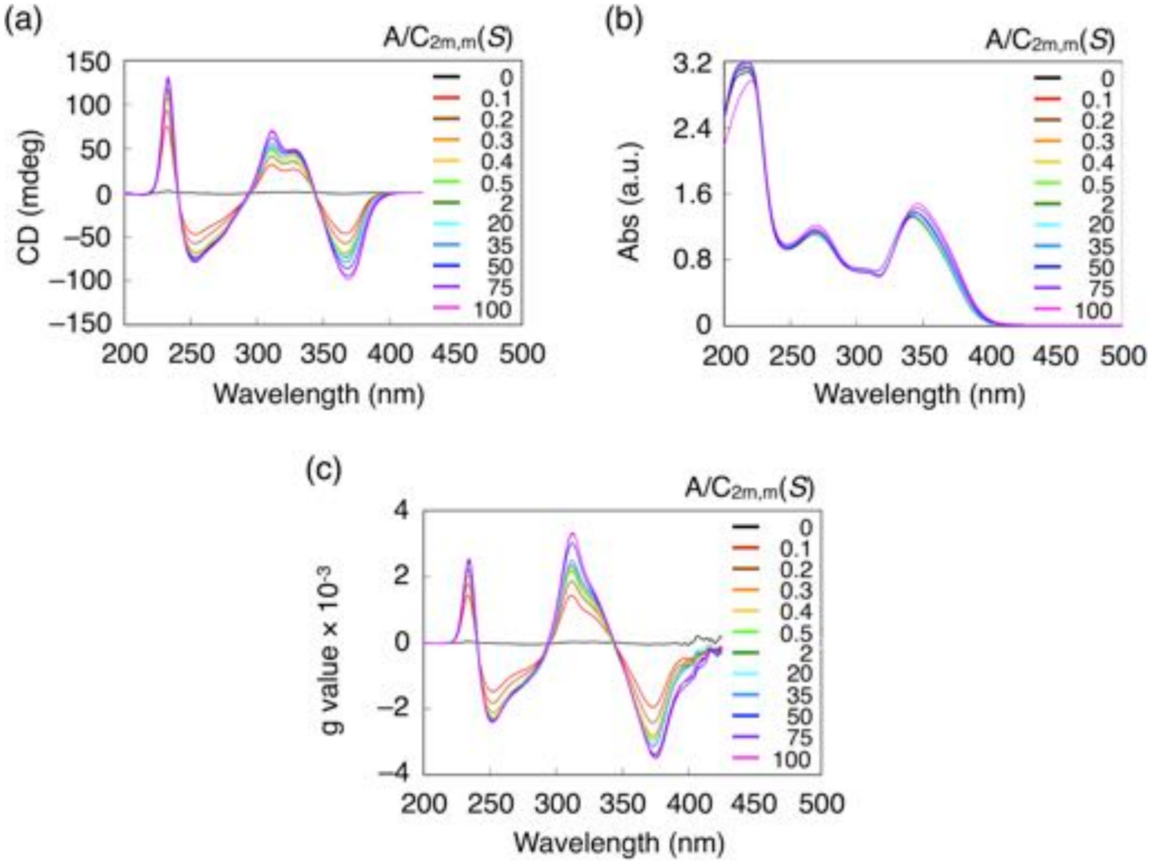

Figure S28. (a) CD, (b) electronic absorption, and (c) g-value spectra for mixtures of **A/C$_{2m,m}$(*S*)** containing various mole fractions of the sergeants at a total concentration of 30 μM at 25 °C in cyclohexane.

4.11. Sergeants and Soldiers Experiments for Mixtures of $A_{-p}/C_3(S)$

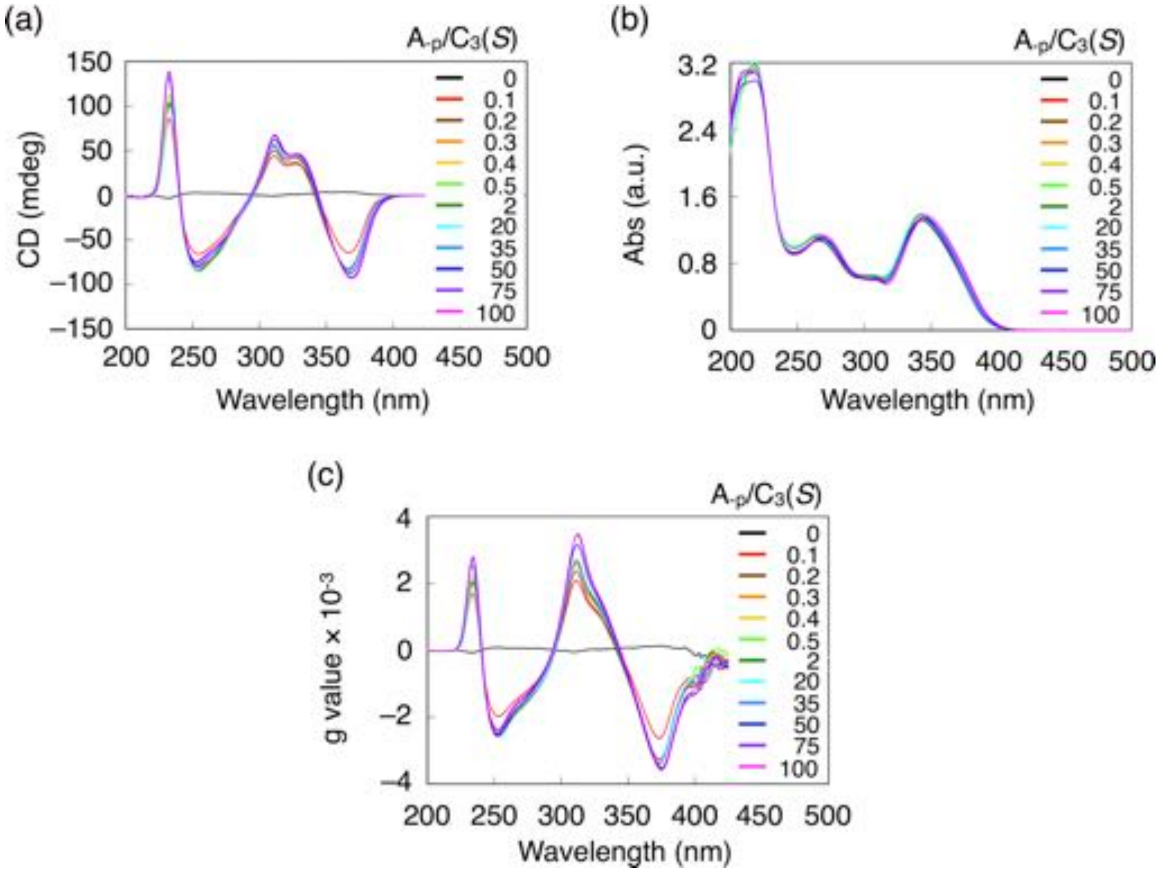

Figure S29. (a) CD, (b) electronic absorption, and (c) g-value spectra for mixtures of $\mathbf{A_{-p}/C_3(}\boldsymbol{S}\mathbf{)}$ containing various mole fractions of the sergeants at a total concentration of 30 μM at 25 °C in cyclohexane.

4.12. Sergeants and Soldiers Experiments for Mixtures of $\mathbf{A_{-m}/C_3}$(*S*)

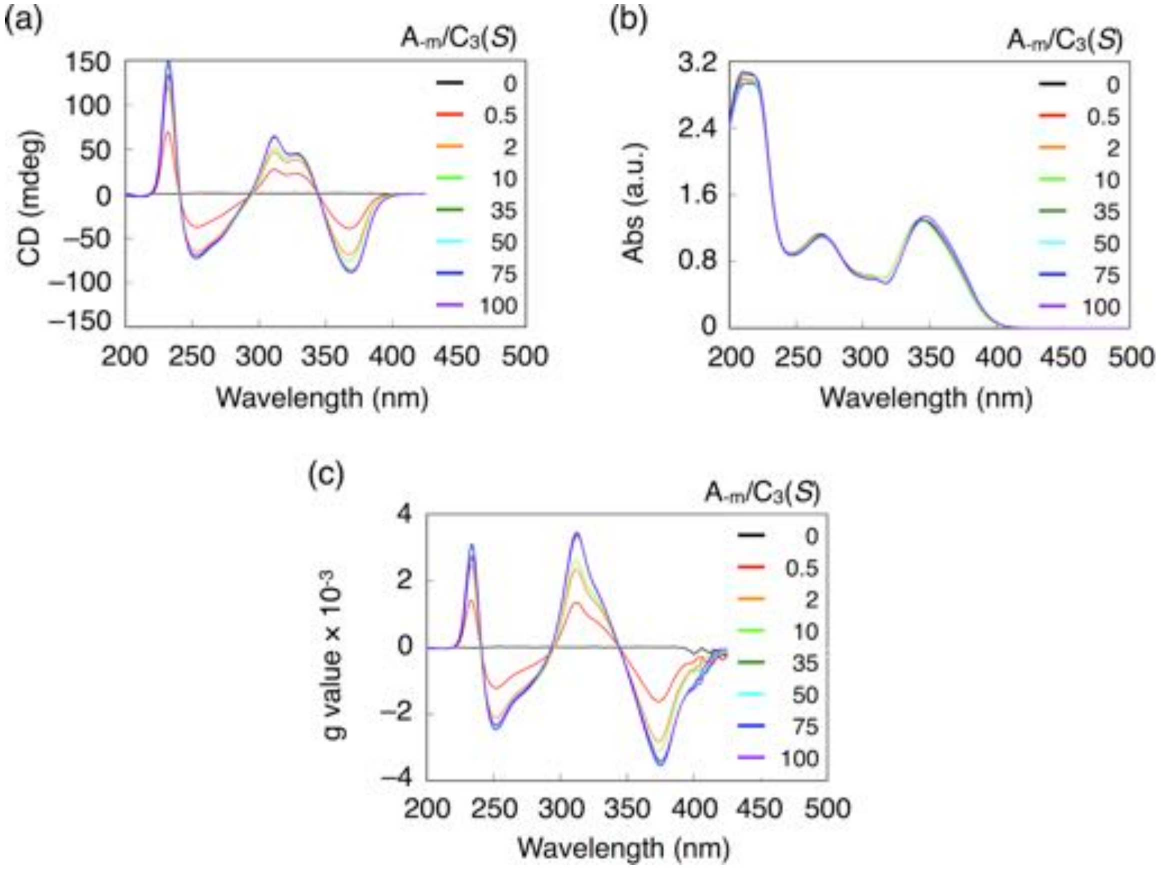

Figure S30. (a) CD, (b) electronic absorption, and (c) g-value spectra for mixtures of $\mathbf{A_{-m}/C_3}$(*S*) containing various mole fractions of the sergeants at a total concentration of 30 μM at 25 °C in cyclohexane.

4.13. Comparison of the Self-assembly Behavior of A, A_{-p}, and A_{-m}

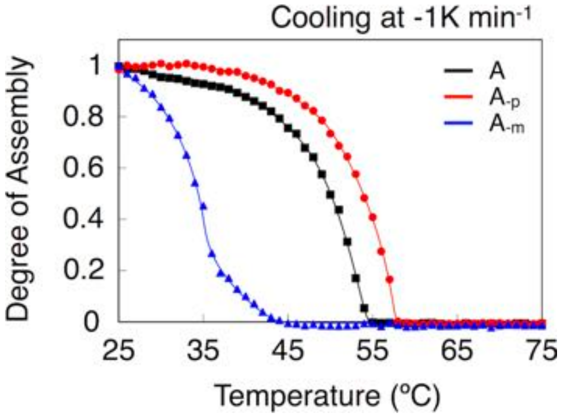

Figure S31. Plots of the degree of assembly of A, A_{-p}, and A_{-m} during the cooling process from 75 °C to 25 °C at a concentration of 30 μM in cyclohexane. The degree of assembly at the individual temperatures was obtained using the following equation and the electronic absorbance at 315 nm.

Degree of Assembly (T °C) = (Abs. (75 °C). – Abs. (T °C)) / (Abs. (75 °C) – Abs. (25 °C))

4.14. Sergeants and Soldiers Experiments for Mixtures of C_{1p}(*S*)/C_3(*R*)

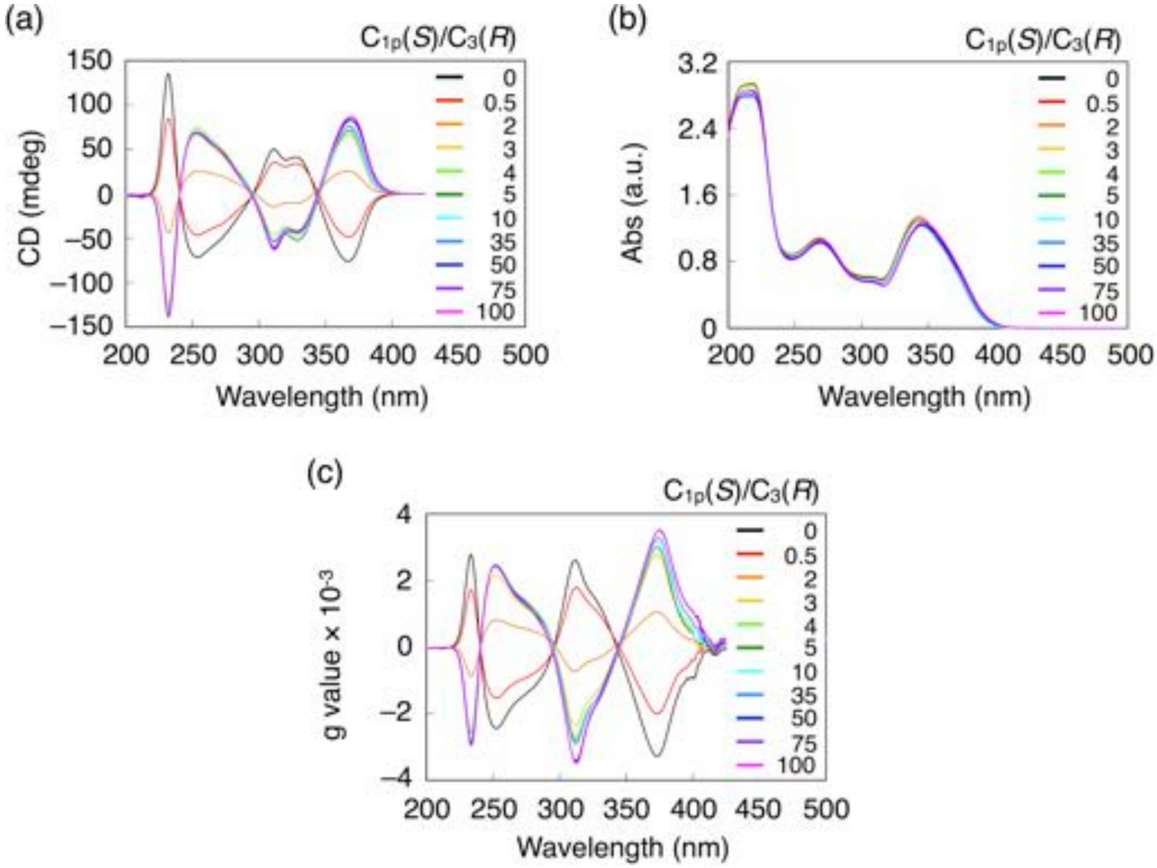

Figure S32. (a) CD, (b) electronic absorption, and (c) g-value spectra for mixtures of **C_{1p}(*S*)/C_3(*R*)** containing various mole fractions of the sergeants at a total concentration of 30 μM at 25 °C in cyclohexane.

4.15. Majority Rule Experiments for Mixtures of C_{1p}(***R***)/C_{1p}(*S*) (1)

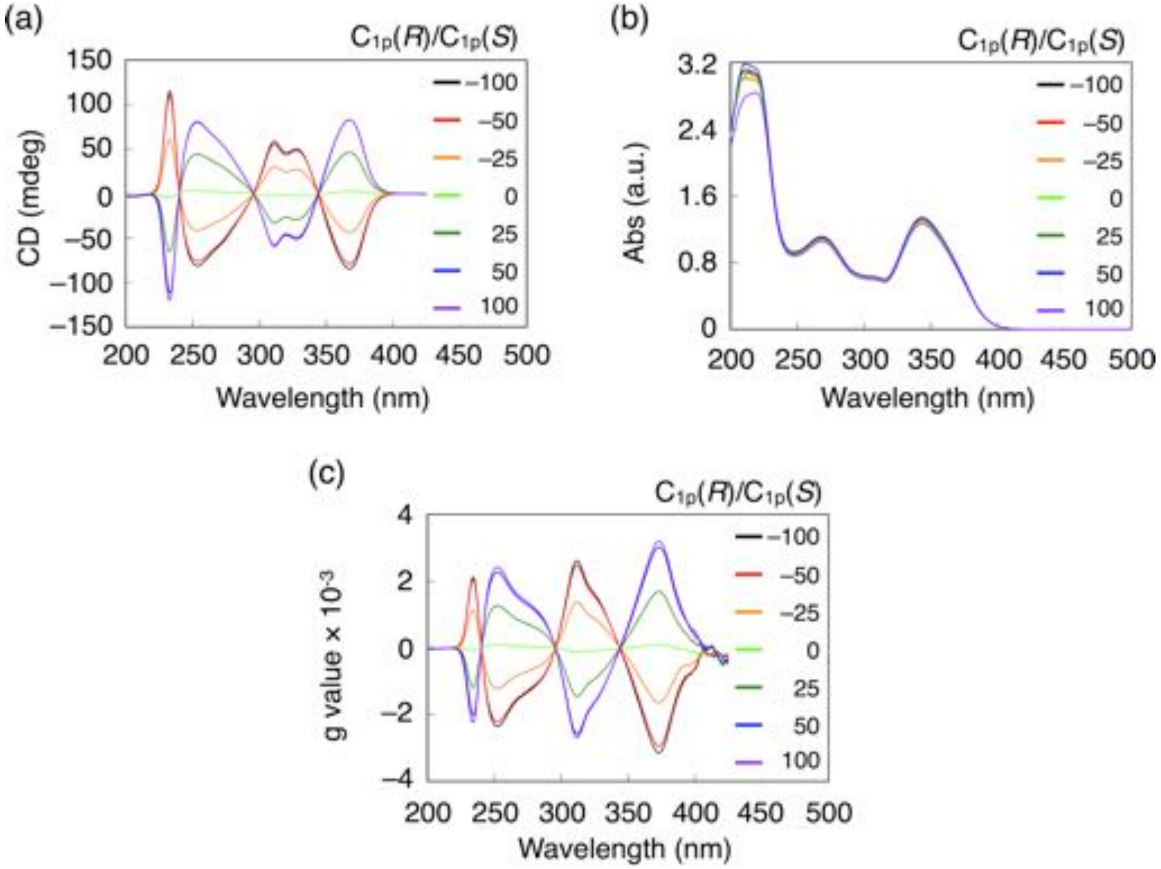

Figure S33. (a) CD, (b) electronic absorption, and (c) g-value spectra for mixtures of **C_{1p}(*R*)/C_{1p}(*S*)** containing various enantiomeric excess of **C_{1p}(*R*)/C_{1p}(*S*)** at a total concentration of 30 μM at 25 °C in cyclohexane.

4.16. Majority Rule Experiments for Mixtures of C_{1p}(*R*)/C_{1p}(*S*) (2)

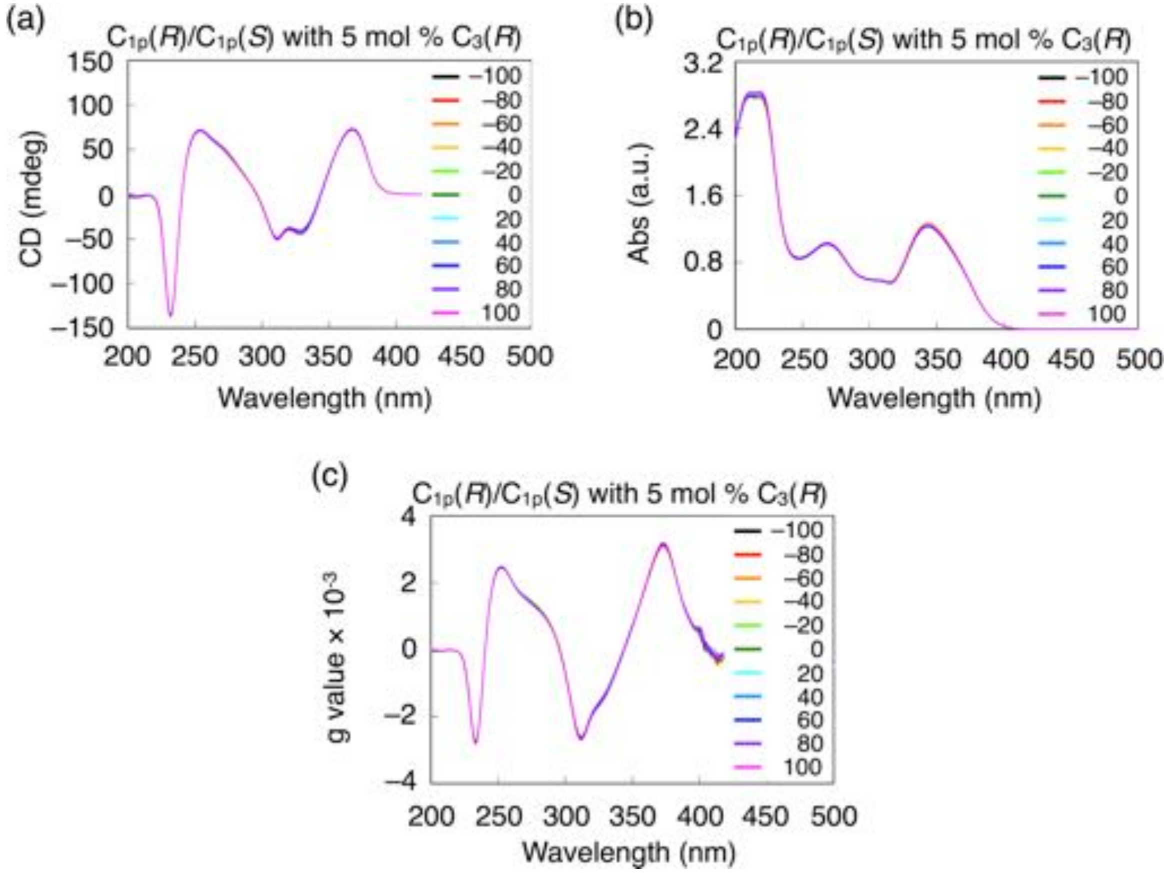

Figure S34. (a) CD, (b) electronic absorption, and (c) g-value spectra for mixtures of **C_{1p}(*R*)/C_{1p}(*S*)** containing various enantiomeric excess of **C_{1p}(*R*)/C_{1p}(*S*)** at a total concentration of 30 μM in the presence of 5 mol% C_3(*R*) at 25 °C in cyclohexane.

4.17. Theoretical Estimation of Number Averaged Degree of Polymerization

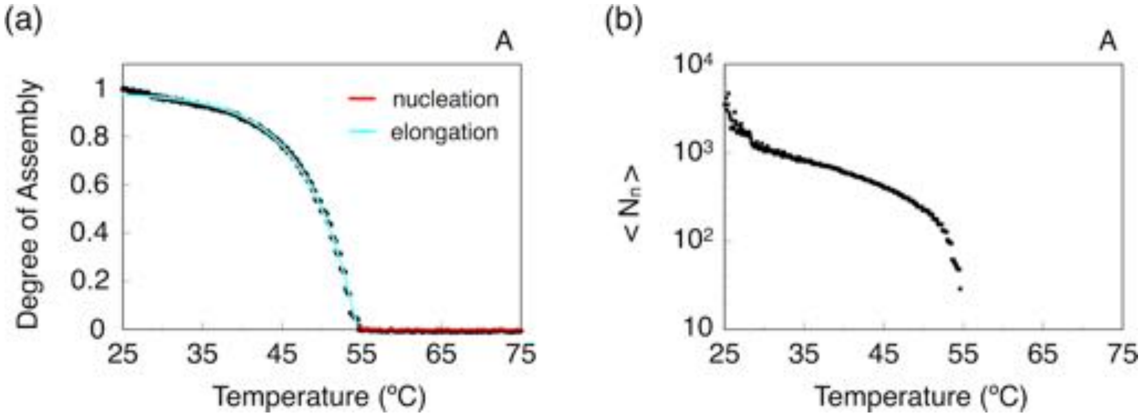

Figure S35. (a) Fit of the nucleation-elongation model and (b) number-averaged degree of polymerization, averaged over all active species, $<N_n>$ for 30 μM **A** in cyclohexane as calculated from equation S.1-3 and based on the temperature-dependent absorption data.[S10]

For elongation regime

$$\phi_n = \phi_{SAT}\left(1 - exp\left[\frac{-h_e}{RT_e^2}(T - T_e)\right]\right) \quad \text{(S.1)}$$

For nucleation regime

$$\phi_n = \phi_{SAT}\left[\sqrt[3]{K_a}exp\left[\left(\frac{2}{3\cdot\sqrt[3]{K_a}} - 1\right)\frac{-h_e}{RT_e^2}(T - T_e)\right]\right]\left(1 - exp\left[\frac{-h_e}{RT_e^2}(T - T_e)\right]\right) \quad \text{(S.2)}$$

Number-averaged degree of polymerization, $<N_n>$

$$< N_n > = \frac{1}{\sqrt{K_a}}\frac{\phi_n}{\phi_{SAT} - \phi_n}$$

h_e: the molecular enthalpy, T_e: the elongation temperature, ϕ_n: the degree of aggregation, ϕ_{SAT}: the parameter, K_a: the dimensionless equilibrium constant of the activation step at T_e.

4.18. Error Ranges of Sergeants and Soldiers Experiments

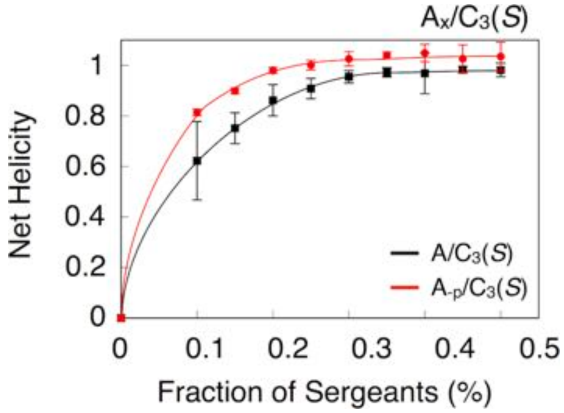

Figure S36. Plots of the net helicity against the fraction of the sergeants for the mixtures of **A/C$_3$(*S*)** and **A$_{-p}$/C$_3$(*S*)**. The error bars in the figure indicate the standard deviations from three experiments performed. The total TPA concentration and the experimental temperature were 30 μM and 25 °C, respectively, for all measurements.

5. References

[S1] N. Berova, L. Di Bari, G. Pescitelli, *Chem. Soc. Rev.*, 2007, **36**, 914.

[S2] S. Ghosh, X.-Q. Li, V. Stepanenko, F. Würthner. *Chem. –Eur. J.*, 2008, **14**, 11343.

[S3] P. Nguyen, L. Douce, R. Ziessel, *Tetrahedron Lett.*, 2002, **43**, 5441.

[S4] H. Tamiaki, K. Ogawa, K. Enomoto, K. Taki, A. Hotta, K. Toma, *Tetrahedron*, 2010, **66**, 1661.

[S5] J. van Gestel, A. R. A. Palmans, B. Titulaer, J. A. J. M. Vekemans, *J. Am. Chem. Soc.*, 2005, **127**, 5490.

[S6] F. Vera, R. M. Tejedor, P. Romero, J. Barbera□, M. B. Ros, J. L. Serrano, T. Sierra, *Angew. Chem., Int. Ed.*, 2007, **46**, 1873.

[S7] R. Abbel, M. Wolffs, R. A. A. Bovee, J. L. J. Dongen, X.Lou, O. Henze, W. J. Feast, E. W. Meijer, A. P. H. J. Schenning, *Adv. Mater.*, 2009, **21**, 597.

[S8] M. M. J. Smulders, P. J. M. Stals, T. Mes, T. F. E. Paffen, A. P. H. J. Schenning, A. R. A. Palmans, E. W. Meijer, *J. Am. Chem. Soc.*, 2009, **132**, 611.

[S9] F. Helmich, M. M. J. Smulders, C. C. Lee, A. P. H. J. Schenning, E. W. Meijer, *Angew. Chem., Int. Ed.*, 2011, **133**, 12238.

[S10] M. M. J. Smulders, A. P. H. J. Schenning, E. W. Meijer, *J. Am. Chem. Soc.*, 2008, **130**, 606.
